# Young women's healthcare screening behaviours and sexual autonomy in Ghana: a spatial distribution and socioeconomic inequality analysis of a large population-based survey

**DOI:** 10.3389/frph.2026.1751165

**Published:** 2026-02-09

**Authors:** Yula Salifu, Williams Walana, Joseph Lasong, Mubaric Yakubu, Eleonora Bakintewuni Wobi, Kwasi Torpey

**Affiliations:** 1 Department of Population, Family and Reproductive Health, School of Public Health, University of Ghana, Legon, Ghana; 2 Department of Clinical Microbiology, School of Medicine, University for Development Studies, Tamale, Ghana; 3 Department of Population and Reproductive Health, University for Development Studies, Tamale, Ghana; 4 Department of Population, Family and Reproductive Health, School of Public Health, C.K.T University of Technology and Applied Sciences, Navrongo, Ghana; 5 Department of Biomedical Laboratory Sciences, School of Allied Health Sciences, University for Development Studies, Tamale, Ghana

**Keywords:** breast cancer screening, cervical cancer screening, HIV testing, inequality, sexual autonomy, spatial analysis, young women

## Abstract

**Introduction:**

In Ghana, disparities in screening behaviours and sexual autonomy persist across socioeconomic and geographic lines. This study examined the spatial distribution, determinants, and socioeconomic inequalities in HIV testing, breast, and cervical cancer screening, and sexual autonomy among young women using nationally representative data.

**Methods:**

Data were drawn from the 2022 Ghana Demographic and Health Survey (GDHS) comprising 1,183 currently partnered women aged 15–24 years. Weighted analyses were performed to estimate screening and autonomy prevalence across regions. Multilevel mixed-effects logistic regression identified predictors of screening behaviours, while socioeconomic inequalities were assessed using the Wagstaff concentration index and Theil's index. Spatial analyses were conducted to map regional variations and clustering patterns.

**Results:**

Nationally, 63.9% of young women had ever tested for HIV, while only 12.1% and 3.6% had undergone breast and cervical cancer screening, respectively. Sexual autonomy prevalence was 77.2%. Education, wealth, health insurance, and media exposure significantly predicted screening uptake. Sexual autonomy independently increased the likelihood of breast (AOR = 2.75; 95% CI: 1.27–5.93) and cervical cancer screening (AOR = 5.45; 95% CI: 1.43–20.73). Spatial maps revealed strong north–south gradients, with higher autonomy and screening uptake clustered in southern and coastal regions (Eastern, Volta, and Central), and markedly lower levels in the northern belt (Northern, North-East, and Savannah). Wealth-related inequalities were pronounced for HIV (CI = 0.288) and breast cancer screening (CI = 0.334), but not for cervical cancer screening. Theil's indices confirmed substantial inequity across region and residence.

**Conclusions:**

Targeted strategies such as expanding school-and-community-based health education for young women, integrating HIV and cancer screening into routine primary and antenatal care, strengthening health insurance coverage for preventive services, leveraging mass media to promote screening awareness, and prioritizing mobile and outreach screening services in northern and rural regions are critical for reducing socioeconomic and spatial inequalities in preventive healthcare among young women in Ghana.

## 1 Introduction

The empowerment of adolescent girls and young women is widely recognized as crucial to global health equity and to achieving the United Nations' Sustainable Development Goals on health (SDG 3) and gender equality (SDG 5) (1, 2). In sub-Saharan Africa, these young women continue to bear a disproportionate burden of preventable diseases. For example, cervical cancer incidence in the region is the highest in the world, largely due to limited screening coverage (3). Likewise, adolescent girls and young women in SSA are at high risk of HIV: in 2022, women and girls accounted for about 63% of new HIV infections in sub-Saharan Africa (4). This disproportionate burden is especially concerning for young women who are married or cohabiting, as they often have constrained sexual autonomy and face high exposure to risk (5, 6). These statistics highlight that simply providing services is not enough; uptake depends on broader social and economic factors. For instance, even in Ghana, factors such as a woman's gender, the cost of care, and the availability of health services have been shown to strongly influence whether and where people seek care (7).

In Ghana, the pattern is equally striking. HIV testing programs (for example, integrating testing into antenatal care) have expanded access, but uptake remains low: only about 31% of Ghanaian women aged 15–24 have ever been tested for HIV (8). Uptake of cancer screening is even lower. National survey data indicate that only about 4%–5% of women have ever had a cervical cancer test (9); the DHS 2022 found screening rates of just 6.3% among urban women and 3.0% among rural women (9). Such figures hide deep inequalities. For example, women in poorer northern or rural regions often face greater distance, cost, and facility barriers that limit their use of screening services, so that preventive services remain concentrated among wealthier urban women (10). These disparities illustrate how structural factors shape young women's preventive health behaviors.

Overlaying these structural barriers is the dimension of individual agency, specifically, women's sexual autonomy. Sexual autonomy is often defined as a woman's capacity to refuse sex or insist on condom use and is theorized to empower women to protect their health (5, 11). Research across sub-Saharan Africa shows that sexual autonomy is associated with sexual health outcomes. For example, women with higher sexual autonomy were more likely to report a recent sexually transmitted infection (6) (perhaps reflecting greater awareness or risk). The link between autonomy and intimate partner violence has also been studied, with mixed findings, highlighting complex interactions (5, 12). However, evidence from Ghana is sparse. It remains unclear how women's sexual autonomy influences their uptake of HIV testing or cancer screening once socioeconomic and geographic factors are accounted for.

However, young, partnered women aged 15–24 years are a priority population because they are sexually active and often experience unequal power dynamics in relationships, which may constrain access to HIV testing and cancer screening (5, 13, 14). Previous studies indicate that disparities in HIV testing and cervical cancer screening are particularly pronounced in this group, emphasizing the need for targeted research (15).

To address these gaps, we use data from the 2022 Ghana Demographic and Health Survey (DHS) to conduct a spatial and multilevel analysis focused on young, partnered women (age 15–24). Specifically, our study will (1) map the geographic distribution and identify clusters of sexual autonomy, HIV testing, and breast and cervical cancer screening; (2) examine how sexual autonomy relates to uptake of these services, controlling for socioeconomic, demographic, and locational factors; and (3) quantify socioeconomic inequalities in screening uptake and decompose the contributions of key determinants. In revealing where screening uptake and autonomy are lowest and by identifying the main social and economic barriers, the study will provide clear evidence for directing resources and designing interventions to improve young women's health.

## 2 Theoretical framework and literature review

The persistent disparities in preventive healthcare utilization among young women in low- and-middle-income countries represent a critical challenge to global health equity and the achievement of Sustainable Development Goals (SDGs) 3 and 5 (16). In Ghana, despite national efforts to improve reproductive health services, young women continue to face substantial barriers to essential screenings including HIV testing, breast cancer screening, and cervical cancer screening (17). This theoretical framework integrates multiple perspectives to develop a comprehensive understanding of the multilevel determinants of healthcare utilization and situates the current study within existing literature.

### 2.1 Theoretical foundations

#### 2.1.1 Andersen's behavioral model

Andersen's Behavioral Model of Health Services Use has served as a foundational framework for understanding healthcare utilization patterns across diverse contexts (18). The model's categorization of predisposing, enabling, and need factors provides a systematic approach to examining determinants of health service use. However, the application of this model in patriarchal contexts like Ghana reveals significant limitations. While Andersen's model effectively captures individual-level characteristics and resource availability, it inadequately addresses the gendered power dynamics that fundamentally shape women's access to healthcare services (19). The model's limited attention to agency and empowerment as critical enabling factors represents a substantial theoretical gap when examining women's health behaviors in contexts where gender norms strongly influence decision-making.

#### 2.1.2 Feminist empowerment theory

Feminist empowerment theories substantially enhance understanding by emphasizing the role of women's agency in health-seeking behaviors (20). Kabeer's conceptualization of empowerment as the ability to make strategic life choices highlights how sexual autonomy, which is defined as women's control over their sexual relations and protection, represents a critical dimension of empowerment that directly influences health behaviors. However, the application of feminist theories in the Ghanaian context must account for the complex interplay between individual agency and structural constraints (5). While sexual autonomy may enable women to seek preventive care, its effectiveness is often mediated by patriarchal norms, economic dependencies, and healthcare system barriers that limit the translation of agency into action (21).

#### 2.1.3 Socioeconomic and geographic determinants of healthcare access

The literature consistently demonstrates that socioeconomic status operates as a fundamental determinant of screening behaviors through multiple pathways (10). Wealthier women benefit from greater financial resources to cover direct and indirect costs of care, higher health literacy, and greater access to health information through media exposure (7). The concentration of screening services among wealthier households reflects what McIntyre et al. (22) have termed the inverse care law, where those most in need of services often have least access. In Ghana, this pattern is exacerbated by the country's pronounced north-south developmental divide, which creates geographic disparities in healthcare infrastructure, educational opportunities, and economic development (17, 23).

The spatial dimension of healthcare access represents a critical area of inquiry. Studies across sub-Saharan Africa have documented substantial regional variations in health service utilization, often reflecting historical patterns of infrastructure development and resource allocation (24). In Ghana, the northern regions consistently demonstrate lower healthcare utilization rates, attributed to factors including greater distances to health facilities, fewer healthcare providers, and limited transportation infrastructure (23). However, the mechanisms through which geographic factors interact with individual characteristics remain inadequately explored. The emerging field of health geography emphasizes that place matters not only as a container of services but as a dynamic producer of health outcomes through environmental, social, and cultural pathways (25).

#### 2.1.4 The complex role of sexual autonomy in health-seeking behaviors

Research on sexual autonomy and health outcomes has yielded complex and sometimes contradictory findings. While greater sexual autonomy has been theorized and linked in some studies to reduced risks of intimate partner violence (IPV) and sexually transmitted infections (STIs) in sub-Saharan Africa (5, 6), the evidence remains mixed and context dependent. In contrast, the relationship between autonomy and preventive healthcare utilization is less straightforward. However, sexual autonomy may predict screening behaviors or not and could highlight the context-dependent nature of this relationship.

This possible differential effect may be understood through the lens of the Health Belief Model (26), which emphasizes how perceived susceptibility, severity, benefits, and barriers influence health behaviors. For breast and cervical cancer screening which are typically voluntary and proactive behaviors, sexual autonomy may enable women to overcome barriers such as embarrassment, fear, or spousal disapproval (27). In contrast, HIV testing for young, partnered women in Ghana often occurs through routinized antenatal care, where individual agency may play a less significant role (8, 28). This distinction may show the importance of considering the specific nature of different screening behaviors rather than treating preventive healthcare as a monolithic category.

#### 2.1.5 Theory of gender and power

The Theory of Gender and Power (29) provide a valuable supplementary framework for understanding how gender-based inequalities in power, labor, and social norms shape health behaviors. The theory's emphasis on the sexual division of labor, sexual division of power, and cathexis (emotional investments) helps explain how gendered expectations influence women's ability to seek preventive care. In the Ghanaian context, where women often bear primary responsibility for domestic work while having limited decision-making power, these gendered structures create significant barriers to healthcare utilization (2).

#### 2.1.6 Capability theory

The Capability Approach developed by Sen (30) offers another relevant perspective by focusing on what people are effectively able to do and be. From this viewpoint, healthcare utilization represents not merely a behavior but a capability that depends on both individual agency and social arrangements. This approach helps explain why two women with similar levels of autonomy may exhibit different screening behaviors based on variations in their capability sets, which include factors such as education, social support, and environmental conditions (30).

#### 2.1.7 Multilevel influences and community context

Furthermore, between-cluster variation in screening behaviors may highlight the importance of community-level factors that extend beyond individual characteristics. Social network theory suggests that health behaviors are influenced not only by individual attributes but by the behaviors and attitudes of others in one's social network (31). In contexts where preventive screenings are uncommon, social norms may discourage utilization even among women with high individual autonomy. The concept of collective efficacy which is defined as social cohesion among neighbors combined with their willingness to intervene for the common good, may also influence screening behaviors (32). Communities with high collective efficacy may develop supportive norms around women's health and create environments that facilitate healthcare utilization. Conversely, in communities with low collective efficacy, even autonomous women may face significant social barriers to seeking care.

#### 2.1.8 Intersectional considerations

An intersectional perspective (33) reveals how multiple social identities and positions combine to shape health experiences and outcomes. Young women in Ghana, for instance, may face the compounded disadvantages of gender, age, geographic location, and often lower socioeconomic status. These intersecting positions may create unique experiences of marginalization that cannot be fully understood by examining any single dimension in isolation. The life course perspective further enriches understanding by emphasizing how health behaviors and outcomes accumulate over time (34). Early experiences with healthcare systems, educational opportunities, and exposure to gender norms may establish trajectories that influence health behaviors throughout adulthood. For young women aged 15–24, this represents a critical developmental period when health behaviors and attitudes are being formed, making this age group particularly important for intervention.

Therefore, this integrated theoretical framework demonstrates that young women's screening behaviors in Ghana may be shaped by a complex interplay of individual agency, household resources, community norms, and structural factors. No single theoretical framework adequately captures this complexity, necessitating an integrated approach that combines insights from healthcare utilization models, feminist theories, ecological perspectives, and intersectional analysis. The current study addresses critical gaps through its examination of both individual and community-level factors, its distinction between different screening behaviors, and its spatial analysis of regional patterns. However, additional research is needed to understand the causal pathways linking autonomy to health outcomes, the role of male partners in screening decisions, and the effectiveness of interventions targeting different levels of influence.

## 3 Data and methods

### 3.1 Study design and data source

This study adopted a cross-sectional analytical design using nationally representative data from the 2022 Ghana Demographic and Health Survey (GDHS). The GDHS is part of the global Demographic and Health Surveys (DHS) Program, implemented in low- and middle-income countries to collect standardized and comparable data on key indicators of population, health, and nutrition. The 2022 GDHS was implemented by the Ghana Statistical Service (GSS) in collaboration with the Ghana Health Service (GHS) and ICF International, with technical and financial support from USAID and other development partners.

The GDHS employed a two-stage stratified cluster sampling design based on the 2021 Ghana Population and Housing Census (PHC). In the first stage, 623 enumeration areas (EAs) were selected with probability proportional to size, stratified by region and place of residence (urban/rural). In the second stage, 25 households per cluster were systematically selected from updated household listings. All eligible women aged 15–49 years who were de facto household members were interviewed using standardized DHS questionnaires.

A total of 14,596 women completed interviews, with a response rate of 98.8%. For this analysis, the sample was restricted to married/cohabiting (currently partnered) women aged 15–24 years with complete data on the study variables, resulting in an analytical weighted sample of 1,183 women. DHS data are publicly available upon registration at https://dhsprogram.com.

### 3.2 Study population

The study population consisted of married/co-habiting (currently partnered) women aged 15–24 years in Ghana. To ensure representativeness, all analyses applied sampling weights, stratification, and cluster adjustments as recommended in DHS analytical guidelines. Cases with missing data (<5%) were excluded using listwise deletion, as the pattern of missingness was random based on Little's MCAR test (*p* > 0.05) (35).

### 3.3 Variables

#### 3.3.1 Outcome variables

Three binary outcome variables were examined, reflecting key preventive health behaviours:

1. HIV Testing: coded as 1 if the woman reported ever having been tested for HIV, and 0 if otherwise.
2. Breast Cancer Screening: coded as 1 if she reported ever screened for breast cancer by a doctor (underwent a clinical breast examination or mammogram) and 0 if otherwise.
3. Cervical Cancer Screening: coded as 1 if she had ever screened for cervical cancer by a doctor [underwent a Pap smear or visual inspection with acetic acid (VIA)] and 0 if otherwise.

Each outcome was modelled independently to identify determinants and assess inequalities in coverage.

#### 3.3.2 Main exposure variable: sexual autonomy

Sexual autonomy was conceptualized as the ability of a woman to make decisions regarding her sexual relations and protection against sexually transmitted infections. It was constructed as a composite index derived from three DHS items:

1. Ability to refuse sex with a partner,
2. Ability to ask the partner to use a condom, and
3. Belief that it is justified to ask for condom use if the partner has an STI.

Each variable was coded 1 (Yes) or 0 (No) and summed to obtain an autonomy score ranging from 0 to 3. The final variable was categorized as:

- No/Low autonomy: scores 0–1
- Moderate/High autonomy: scores 2–3

This index aligns with prior DHS-based studies assessing women's empowerment in reproductive decision-making (5, 12).

#### 3.3.3 Explanatory variables

Covariates were selected and categorized according to Andersen's Behavioural Model of Health Services Use (18), which conceptualizes healthcare utilization as being determined by predisposing, enabling, and need factors.

1. Predisposing Factors - Characteristics that exist prior to illness that predispose individuals to use services:
  Demographic: Age
  Social Structure: Educational attainment, employment status
  Health Beliefs: Exposure to mass media (radio, television, newspaper), internet use
2. Enabling Factors—Resources that facilitate or impede service use:
  Personal/Family Resources: Household wealth index, health insurance coverage
  Community Resources: Place of residence (urban/rural), distance to health facility, region
3. Need Factors—Illness levels that generate need for healthcare services:
  Perceived Need: Self-reported health status
  Evaluated Need: Parity, contraception method.0
  Categorization followed DHS conventions to maintain comparability across surveys.

### 3.4 Statistical analysis

#### 3.4.1 Data management and weighting

Analyses were conducted using Stata version 17.0/MP (Stata Corp, College Station, TX) and R version 4.3.2. The analytical framework included descriptive statistics, proportion distributions, multivariable modelling, inequality assessment and spatial distribution.

To account for the complex sampling design, all analyses incorporated stratification, clustering, and sampling weights. The DHS individual sampling weight (v005) was divided by 1,000,000 before use. In Stata 17, the survey design was declared using the “svy” command. All descriptive and inferential statistics were adjusted for the design effect using Taylor linearization. Weighted estimates ensured national representativeness, while standard errors were corrected for clustering at the PSU level.

#### 3.4.2 Descriptive analysis

Weighted frequencies and percentages summarized background characteristics and outcomes. Weighted proportions of the screening outcomes across and explanatory variables were also computed, adjusting for clustering and stratification effects.

#### 3.4.3 Multivariable multilevel mixed-effects logistic regression

Given the hierarchical structure of the GDHS data, with individuals (level 1) nested within clusters or enumeration areas (level 2), two-level mixed-effects logistic regression models were fitted for each outcome. This approach accounted for both individual and community-level heterogeneity in determining the predictors of the outcomes (HIV testing, Breast and Cervical cancer screening).

The general model specification was:

$$\log\;\mathrm{it}(P_{ij})=\beta_{0}+\beta_{1}X_{ij}+\gamma Z_{j}+u_{j}$$

where logit (*P_ij_*) = log-odds of the outcome for individual *i* in cluster *j*; *P_ij_* = probability of the outcome for individual *i* in cluster *j; β*_0_ = overall intercept (fixed effect); *β*_1_ *X_ij_* = vector of coefficients and individual-level covariates (fixed effects) (sexual autonomy, education, wealth, etc); *u_j_* = random intercept for cluster *j*, assumed normally distributed with mean 0 and variance; and *Z_j_* = cluster-level covariates (residence, region).

Two sequential models were estimated:

1. Null model: This had no covariates and estimated baseline variance.
2. Full model: This included all individual and community-level variables.

#### 3.4.4 Model fitness assessment

Model fit was assessed using the Akaike Information Criterion (AIC), Bayesian Information Criterion (BIC), and log-likelihood ratio tests.

#### 3.4.5 Cluster-level variation estimation

Cluster-level variation was quantified using interclass correlation, median odds ratio and proportional change variance.

#### 3.4.6 Intraclass correlation coefficient (ICC)

This measures the proportion of the total variance in the outcome that is attributable to the clustering structure.

$$\mathrm{ICC}=\frac{\sigma_{u}^{2}}{\sigma_{u}^{2}+\frac{\pi^{2}}{3}}\;;\;\;\;\sigma_{u}^{2}=\mathrm{between}\;\;\mathrm{cluster}\;\mathrm{variance}$$

where:

- $\sigma_{u}^{2}$ = between-cluster variance (the variance of the cluster-level random intercepts).
- *π* = the mathematical constant Pi (approximately 3.14159). The term $\frac{\pi^{2}}{3}$ represents the variance of the standard logistic distribution, which is the assumed level-1 (individual) variance in a logistic multilevel model.

#### 3.4.7 Median odds ratio (MOR)

It represents the median value of the odds ratio between two randomly chosen individuals with identical covariates from two different clusters, where one is from a higher-risk cluster and the other from a lower-risk cluster

$$\mathrm{MOR}=\exp\;\left(0.95\times \sqrt{\sigma_{u}^{2}}\right)$$

where:

- $\sigma_{u}^{2}$ = between-cluster variance (the variance of the cluster-level random intercepts).

#### 3.4.8 Proportional change in variance (PVC)

PVC measures how much of the between-cluster variance is explained by adding covariates to the model. It ranges from 0% to 100%, where: 0% means covariates explain none of the between-cluster variance and 100% means covariates explain all the between-cluster variance

$$\mathrm{PVC}=\left(\frac{\sigma_{u,\mathrm{null}}^{2}-\sigma_{u,\mathrm{full}}^{2}}{\sigma_{u,\mathrm{null}}^{2}}\right)\times 100\text{\%}$$

where:

- $\sigma_{u,\mathrm{null}}^{2}$ = between-cluster variance from the null model (a model with no individual-level or household-level covariates, only the random intercept).
- $\sigma_{u,\mathrm{full}}^{2}$ = between-cluster variance from the full model (the model including all covariates).

#### 3.4.9 Model performance and collinearity assessment

Model performance was further evaluated using McFadden's pseudo-*R*^2^ and receiver operating characteristic of the area under curve (ROC-AUC) statistics to assess predictive accuracy. ROC-AUC values >0.7 indicated good model accuracy. Multicollinearity was checked using Generalized Variance Inflation Factors (GVIFs), with GVIF >10 indicating high collinearity. However, all the models showed GVIF <10.

#### 3.4.10 Inequality analysis

Socioeconomic inequalities in screening coverage were examined using three complementary approaches: rich–poor ratio, Concentration Index (CI), and Theil's T index.

#### 3.4.11 Concentration Index (CI)

The CI measures the extent of socioeconomic inequality in a health variable [18]:

$$\mathrm{The}\;\mathrm{concentration}\;\mathrm{index}\;\mathrm{is}\;\;\mathrm{defined}\;\mathrm{as}\;\mathrm{CI}=\frac{2}{\mu }\times \mathrm{cov}(h,r)$$

where:

*μ* = mean of the health variable
*h* = health variable(screening outcome, 0/1)
*r* = fractional rank of individuals by wealth index
cov(*h*,*r*) = covariance between *h* and *r*.

The concentration curve was constructed by plotting the cumulative proportion of the population of young women, ranked from the poorest to the richest according to the household wealth index, against the cumulative proportion of the health service coverage (HIV testing, cervical and breast cancer screening). The 45-degree line represents the Line of Equality. If the concentration curve lies exactly on this line, it signifies the absence of socioeconomic inequality—the health service is equally utilized by individuals across all wealth levels. When the concentration curve lies below the line of equality (the 45-degree line), it indicates that the coverage of the service is disproportionately concentrated among individuals with higher socioeconomic status; conversely, a curve lying above the line of equality reflects a pro-poor distribution (36).

The curves were generated using the “Lorenz” package in Stata, while the concentration index (CI) was computed with the “conindex” command (37). The CI represents twice the area between the concentration curve and the line of equality, and it quantifies the degree of socioeconomic-related inequality in the distribution of testing and screening coverage. In this study, the CI was used to assess the relationship between the cumulative proportion of young women ranked by wealth and the cumulative proportion of coverage for each testing/screening service. The CI values range from −1 to +1, where values close to zero indicate perfect equality, positive values denote a pro-rich inequality, and negative values suggest a pro-poor inequality (36). For binary outcomes, Wagstaff's normalization was applied to ensure boundedness. Standard errors were bootstrapped (1,000 replications) to test significance. Subgroup decomposition of the CI by urban–rural residence, education and region was performed to identify within- and between-group disparities (36). Inequality between the groups was considered statistically significant with *p*-values less than 5%.

#### 3.4.12 Theil's T Index

To assess absolute inequality, Theil's T index (38) was computed:

$$T=\overset{n}{\underset{i=1}{\sum }}\;(\;p_{i}\cdot r_{i}\cdot \ln\;(r_{i}))$$

where:

*p_i_* = population share of subgroup *i*
*r_i_* = ratio of subgroup prevalence *i* to overall population prevalence.

Theil's index ranges from 0 (perfect equality) to higher values indicating greater levels of relative inequality. It was further decomposed into within-group and between-group components to explore sources of disparity. Results were multiplied by 1,000 for interpretability. This index was computed to measure inequality concerning residence, geographical location and education.

#### 3.4.13 Rich–poor ratio

The rich–poor ratio was calculated as:

$$\mathrm{Rich}-\mathrm{poor}\;\;\mathrm{ratio}=\frac{\mathrm{Prevalenc}e_{\mathrm{richest}}}{\mathrm{Prevalenc}e_{\mathrm{poorest}}}$$

This simple measure complements the CI and Theil's index by providing a direct comparison between the extremes of the wealth distribution.

#### 3.4.14 Spatial analysis

Spatial heterogeneity in screening coverage and sexual autonomy was visualized using choropleth maps in R (version 4.3.2) with dplyr, sf, ggplot2, tidyr, stringr, and viridis and tmap packages. Regional prevalence estimates were calculated using:

$$\hat{\;p_{j}}=\frac{\sum w_{ij}y_{ij}}{\sum w_{ij}}$$

where:

- $\hat{\;p_{j}}$ = weighted prevalence
- *w_ij_* = sample weight
- *y_ij_* = binary outcome for individual *i* in region *j*

Administrative shapefiles from the Ghana Statistical Service and GADM (Global Administrative Database) were merged with survey data to display regional variation.

### 3.5 Ethical considerations

The GDHS protocol received ethical clearance from the Ghana Health Service Ethical Review Committee and the ICF Institutional Review Board. Written informed consent was obtained from all respondents. For participants aged 15–17 years, both parental consent and adolescent assent were secured. This study utilized anonymized secondary data publicly available from the DHS Program; hence, no additional ethical approval was required. All analyses followed the STROBE reporting guidelines for cross-sectional studies.

## 4 Results

### 4.1 Participant characteristics

Table 1 presents the weighted background characteristics of 1,183 young women included in the study. Most participants (77.2%) reported moderate to high sexual autonomy, while nearly two-thirds (63.1%) were not using any contraceptive method. The majority (84.2%) were aged 20–24 years, and two-thirds (66.7%) had attained secondary education, whereas only 0.9% had higher education. About 67% were currently working, and 59.4% resided in rural areas. Moreover, about half of the participants were in the poorer to middle wealth quintiles (43.8%), and 7% were in the richest category. Most women (79.7%) rated their health as good, and 73.2% reported that distance to a health facility was not a major problem. Nearly seven in ten (69.6%) had one or two children, and 92.1% were enrolled in a health insurance scheme. However, a vast majority (92.4%) did not read newspapers, and 42.5% did not listen to the radio at all. Also, over half (55%) reported watching television at least once a week, and 18.5% used the internet almost daily. Regional distribution showed that participants were most concentrated in the Ashanti (15.1%) and Northern (13.9%) regions, with the least representation from Ahafo (2.2%) and Western North (2.3%). Concerning health screening behaviour, 63.9% had ever tested for HIV, while breast and cervical cancer screening uptake remained low, in that; only 12.1% and 3.6%, respectively, had ever undergone these screenings (Table 1).

**Table 1:** Weighted distribution of participant characteristics (*N* = 1,183).

Variable	Category	Weighted count (n)	Weighted percentage (%)
Sexual autonomy	Low/No	270	22.8
	Moderate/High	913	77.2
Contraception Method	No method	746	63.1
	Hormonal	299	25.3
	Non-hormonal	137	11.6
Age	15–19	187	15.8
	20–24	995	84.2
Education	No education	190	16.0
	Primary	193	16.4
	Secondary	789	66.7
	Higher	11	0.9
Current Working status	No	393	33.2
	Yes	790	66.8
Residence	Urban	480	40.6
	Rural	702	59.4
Wealth	Poorest	344	29.1
	Poorer	256	21.7
	Middle	262	22.1
	Richer	238	20.2
	Richest	83	7.0
Self-reported Health status	Good	943	79.7
	Bad	240	20.3
Distance problem to health facility	Not a big problem (≤30 min)	866	73.2
	Big problem (>30 min)	317	26.8
Total children ever born	No child	262	22.2
	1–2 children	824	69.6
	3+ children	97	8.2
Health insurance	No	93	7.9
	Yes	1,090	92.1
Frequency to Read newspaper	Not at all	1,093	92.4
	<1/week	75	6.3
	≥1/week	15	1.2
Frequency to Listen radio	Not at all	503	42.5
	<1/week	259	21.9
	≥1/week	421	35.6
Frequency to Watch TV	Not at all	375	31.7
	<1/week	158	13.3
	≥1/week	650	55.0
Frequency to Internet use	Not at all	780	66.0
	<1/week	49	4.1
	≥1/week	135	11.4
	Almost daily	219	18.5
Region	Western	66	5.6
	Central	111	9.4
	Greater Accra	101	8.5
	Volta	43	3.6
	Eastern	76	6.4
	Ashanti	179	15.1
	Western North	27	2.3
	Ahafo	26	2.2
	Bono	33	2.8
	Bono East	63	5.3
	Northern	164	13.9
	North-East	58	4.9
	Upper East	93	7.9
	Upper West	53	4.5
	Savannah	42	3.6
	Oti	47	3.9
Ever tested for HIV	No	427	36.1
	Yes	756	63.9
Breast cancer screening	N0	1,040	87.9
	Yes	143	12.1
Cervical cancer screening	No	1,140	96.4
	Yes	43	3.6

### 4.2 Distribution of participant characteristics by screening outcomes

Table 2 shows the distribution of participant characteristics by screening outcomes. Women with secondary education accounted for most screening participation, with about 75% for HIV testing and over 70% for both breast and cervical cancer screening. Similarly, participation was highest among women in the richer and richest wealth quintiles, while urban–rural differences were also evident. For breast and cervical cancer screening, uptake was notably higher among urban women (60.1% and 64.3%, respectively) compared to their rural counterparts (39.9% and 35.7%). Moreover, women who watched TV or used the internet almost daily were more likely to have undergone screening than those with no exposure (Table 2).

**Table 2:** Weighted percentage distribution of background characteristics among women by screening outcomes.

Variable	Category	HIV testing (%)	Breast cancer screening (%)	Cervical cancer screening (%)
Sexual Autonomy	Low/No autonomy	18.6	7.2	5.2
	Moderate/High autonomy	81.4	92.8	94.8
Method Use	No method	60.3	52.1	77.1
	Hormonal method	27.4	32.3	14.4
	Non-hormonal method	12.3	15.5	8.5
Age Group (years)	15–19	12.2	7.3	8.4
	20–24	87.8	92.7	91.6
Education Level	No education	9.4	4.6	14.1
	Primary	14.1	12.5	14.2
	Secondary	75.1	80.5	71.7
	Higher	1.4	2.4	—
Working Status	Not working	32.2	29.2	25.6
	Working	67.8	70.8	74.4
Residence	Urban	44.6	60.1	64.3
	Rural	55.4	39.9	35.7
Wealth Quintile	Poorest	21.4	13.8	11.7
	Poorer	21.8	15.8	25.1
	Middle	26.0	25.5	45.9
	Richer	21.5	25.9	12.9
	Richest	9.3	18.9	4.4
Self-rated Health	Good	79.3	81.3	82.1
	Bad	20.7	18.7	17.9
Distance to Facility	Not a big problem	77.6	77.4	76.0
	Big problem	22.4	22.6	24.0
Number of Children	No child	11.1	15.2	18.0
	1–2 children	79.6	78.6	81.2
	3 or more children	9.4	6.2	0.7
Health Insurance	No	3.9	9.2	1.4
	Yes	96.1	90.8	98.6
Reads Newspaper	Not at all	90.4	87.2	92.3
	<Once/week	7.8	11.7	7.7
	≥Once/week	1.8	1.2	—
Listens to Radio	Not at all	37.3	36.6	23.9
	<Once/week	21.9	18.7	16.8
	≥Once/week	40.8	44.7	59.3
Watches TV	Not at all	26.2	17.6	12.4
	<Once/week	13.4	10.7	13.9
	≥Once/week	60.4	71.6	73.7
Internet Use	Not at all	62.5	51.1	51.1
	<Once/week	4.6	2.8	8.7
	≥Once/week	12.0	18.1	21.9
	Almost daily	20.9	27.9	18.3
Region	Western	6.2	11.0	—
	Central	11.4	13.5	14.4
	Greater Accra	8.9	12.7	—
	Volta	4.9	7.7	5.2
	Eastern	9.0	11.1	—
	Ashanti	16.7	9.3	27.4
	Western North	2.3	0.8	0.9
	Ahafo	2.0	3.3	2.5
	Bono	2.9	1.9	1.3
	Bono East	4.7	2.4	3.2
	Oti	4.2	2.8	4.6
	Northern	9.0	8.7	27.5
	Savannah	1.3	1.2	—
	North-East	3.9	6.0	5.8
	Upper East	8.6	4.9	3.6
	Upper West	4.1	2.7	3.5

### 4.3 Spatial distribution of sexual autonomy rate among young women in Ghana

**Figure 1:**
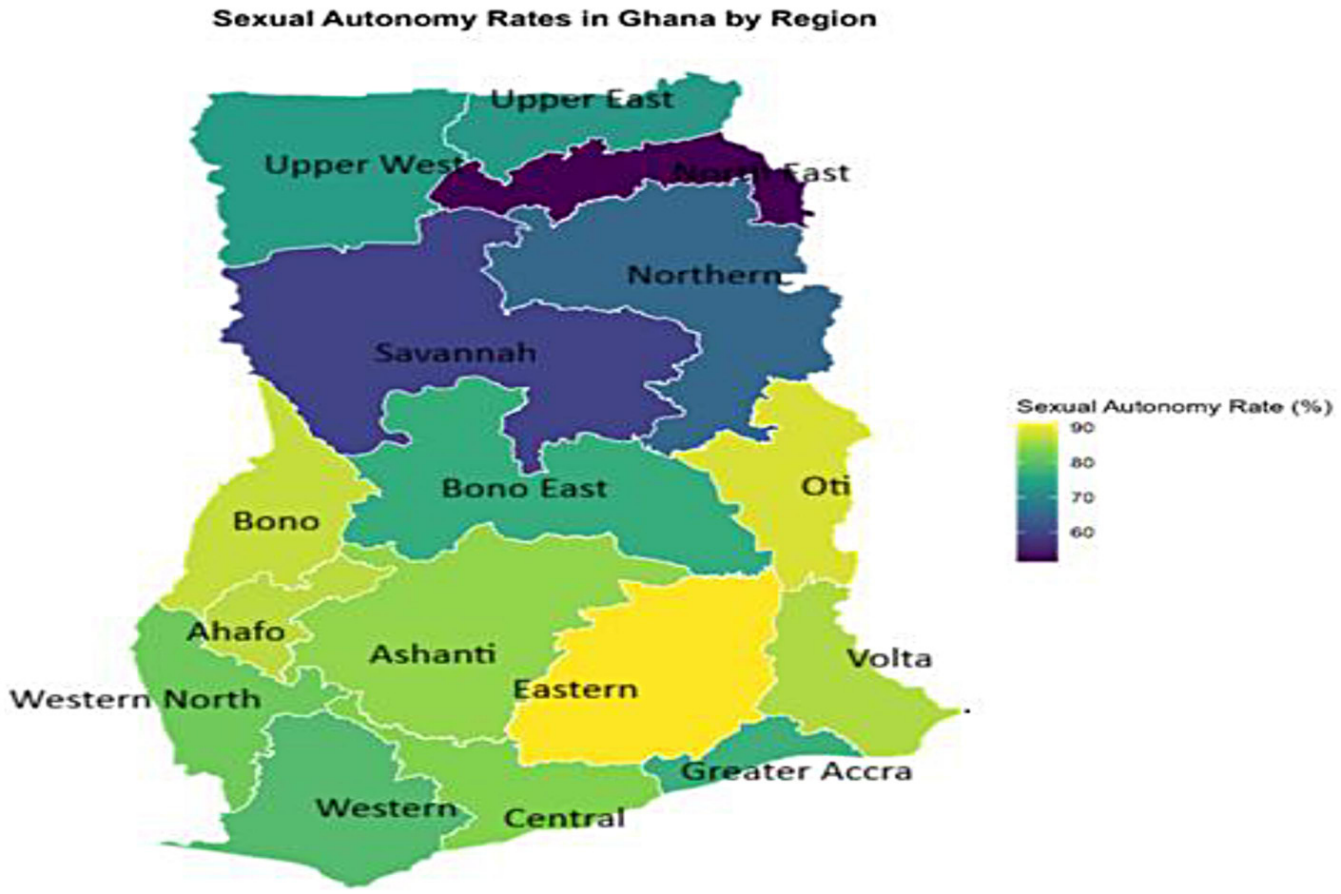
Spatial distribution of sexual autonomy rates among young women in Ghana.

Figure 1 depicts the spatial distribution of women's ability to make independent decisions regarding sexual activity. The gradient shows a strong north–south contrast, with autonomy being highest in the southern and coastal regions and lowest in the northern belt. Hotspot clusters indicate high autonomy with bright yellow and light green colouration, which were found predominantly in the Eastern (91.34%), Oti (88.94%), Bono (87.64%), Ahafo (86.97%), and Volta (86.36%) regions. Spatially, this forms a southern coastal and middle-belt empowerment corridor. However, cold-spot clusters indicate low sexual autonomy with dark blue to purple colouration, which were found mostly clustered around the Northern (64.98%), North-East (51.49%), and Savannah (59.41%) regions. The clustering indicates positive spatial autocorrelation, revealing that low-autonomy regions are spatially adjacent (Figure 1).

### 4.4 Spatial distribution of HIV testing rates among young women in Ghana

**Figure 2:**
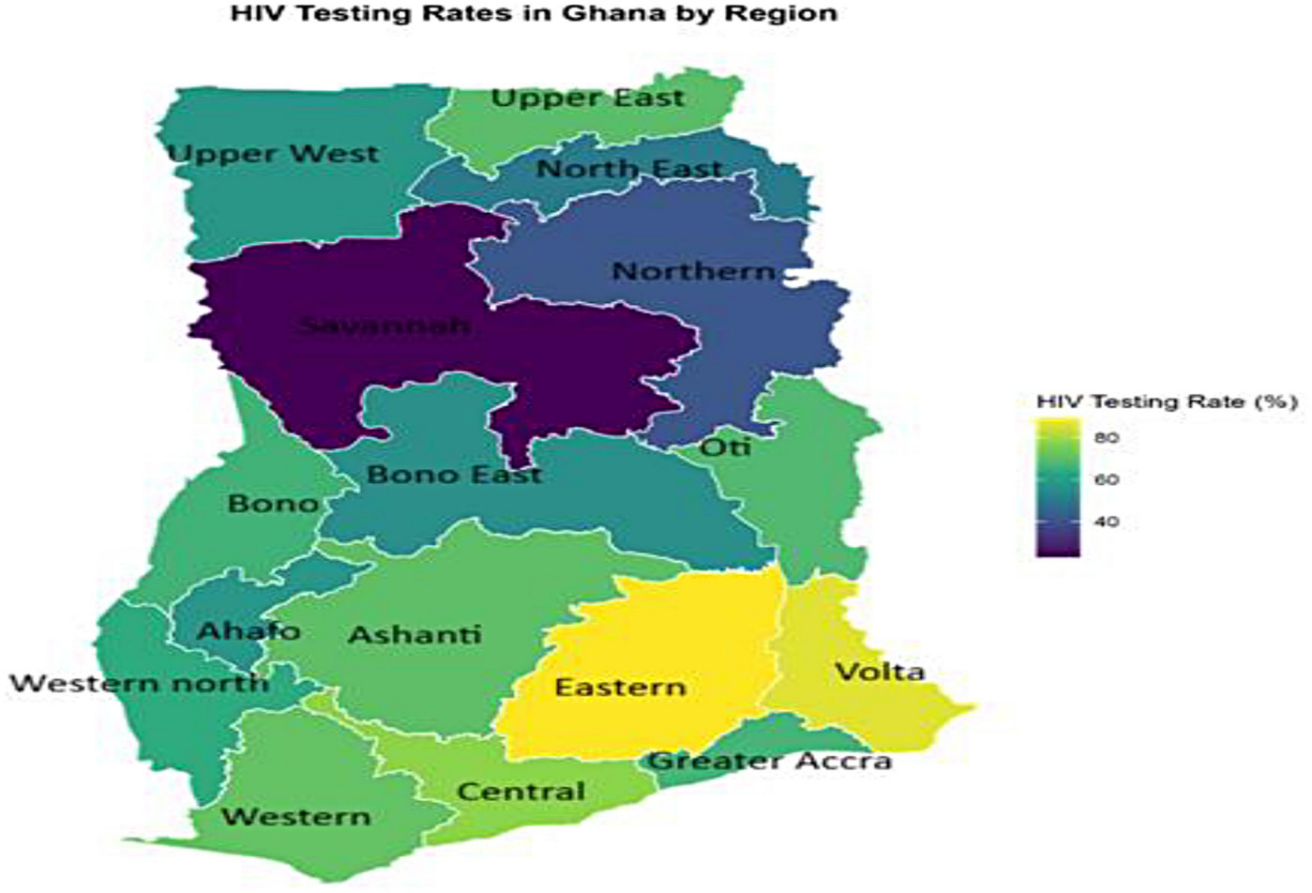
Spatial distribution of HIV testing rates among young women in Ghana.

Figure 2 illustrates regional differences in HIV testing coverage among young women of reproductive age. The colour gradient shows yellow and light-green areas with high testing coverage, and dark blue–purple areas with low coverage. Hotspot clusters indicate high HIV testing burden dominated by Eastern (89.35%), Volta (86.08%) and Central regions (77.70%). Testing rates exceed 70% in these regions, forming a coastal–southern testing cluster. For the cold-spot cluster, which indicates low HIV testing, the Northern (41.27%), Savannah (22.86%), and North-East regions (50.45%) were predominant. The pattern demonstrates geographical inequality, with a clear north–south divide (Figure 2).

### 4.5 Spatial distribution of cervical cancer screening rates among young women in Ghana

**Figure 3:**
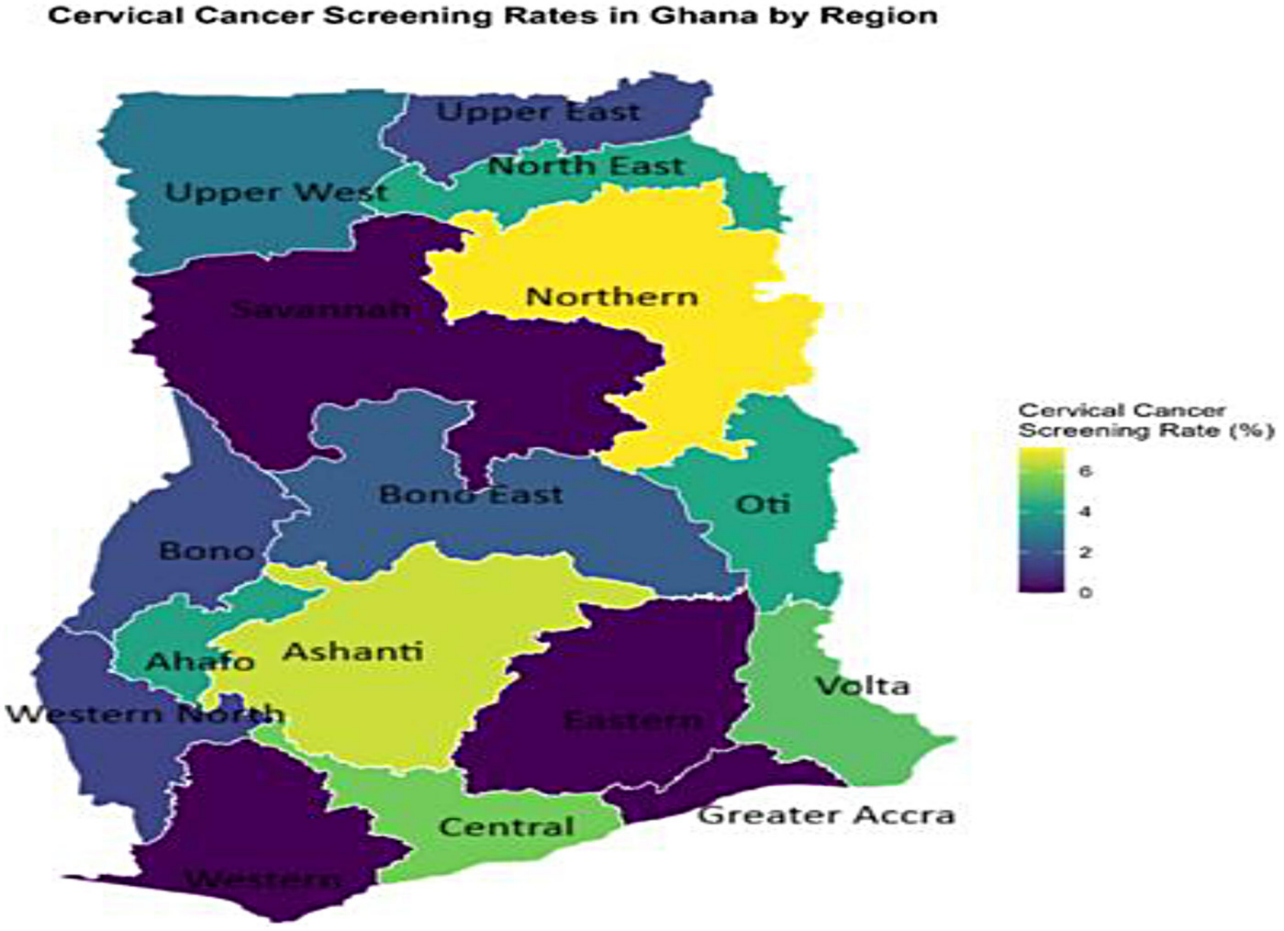
Spatial distribution of cervical cancer screening rates among young women in Ghana.

Figure 3 shows the geographical spread of cervical cancer screening among women aged 15–24 years. The pattern is nearly uniformly polarized, with some high coverage and extreme deficits in both northern and southern regions. Hotspot clusters indicated that Northern, Ashanti and Central regions contributed most of the higher cervical screening rates of about 4%–6%. Moreover, cold-spot clusters encompassed Savannah, Eastern, Greater Accra and Western regions with screening coverage below 2% (Figure 3).

### 4.6 Spatial distribution of breast cancer screening rates among young women in Ghana

**Figure 4:**
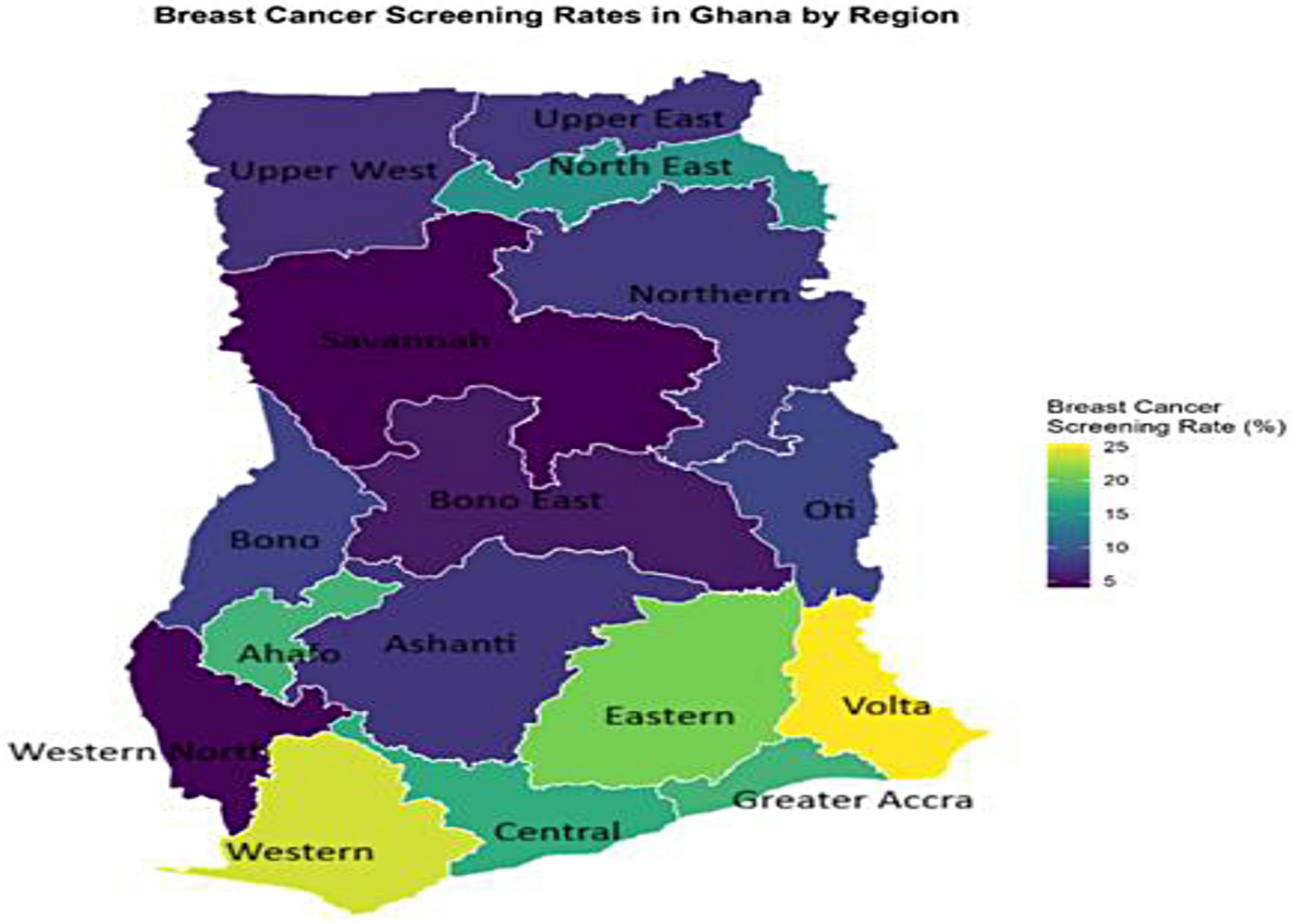
Spatial distribution of breast cancer screening rates among young women in Ghana.

Figure 4 depicts a highly significant geographical pattern in breast cancer screening practices among young women in Ghana. The highest breast cancer screening rates are concentrated in the southern parts of the country, forming a distinct belt of high uptake. The Volta (25.43%), Western (23.95%), Eastern (20.89%), Greater Accra (18.00%) and Ahafo (17.99%) regions had the highest uptake of breast cancer screening. In contrast, the lowest screening rates are found in the northern and middle-belt regions. The Savannah (4.08%), Western North (4.26%) and Bono East (5.53%) regions had the lowest uptake of breast cancer screening (Figure 4).

### 4.7 Predictors of screening practices among young women in Ghana

Table 3 summarizes the fixed and random effects for determinants of HIV testing, breast cancer, and cervical cancer screening among young women in Ghana. The models demonstrated acceptable fit with McFadden's *R*^2^ values of 0.24, 0.16, and 0.27, respectively. The intra-cluster correlation coefficients (ICC) were 0.21, 0.18, and 0.35, suggesting moderate community-level clustering. The Median Odds Ratios (MORs) were 1.98, 1.76, and 2.45 for HIV, breast, and cervical cancer screening models, respectively, indicating substantial between-cluster variability even after accounting for individual-level factors. Particularly, the MOR of 2.45 in the cervical cancer model indicates that, if two women with identical characteristics lived in different clusters, the woman in the higher-risk community would have 2.45 times higher odds of screening participation, reflecting strong contextual effects.

**Table 3:** Multivariable multilevel mixed effects model of predictors of screening practices among young women in Ghana.

Predictor	Category	HIV testing AOR [95% CI]	*p*-value	Breast cancer screening AOR [95% CI]	*p*-value	Cervical cancer screening AOR [95% CI]	*p*-value
Sexual autonomy	Low/No	1 (ref)		1 (ref)		1 (ref)	
	Moderate/High	1.24 [0.79–1.95]	0.358	2.75 [1.26–5.97]	0.011	5.45 [1.44–20.67]	0.013
Contraception Method	No method	1 (ref)		1 (ref)		1 (ref)	
	Hormonal	0.72 [0.48–1.08]	0.114	1.67 [0.95–2.93]	0.074	0.39 [0.14–1.11]	0.079
	Non-hormonal	0.63 [0.37–1.08]	0.094	1.11 [0.52–2.39]	0.788	0.25 [0.08–0.85]	0.026
Age	15–19	1 (ref)		1 (ref)		1 (ref)	
	20–24	1.21 [0.79–1.86]	0.383	2.21 [0.93–5.24]	0.073	2.29 [0.62–8.49]	0.217
Education	No education	1 (ref)		1 (ref)		1 (ref)	
	Primary	1.41 [0.82–2.44]	0.212	2.72 [0.85–8.66]	0.090	1.13 [0.29–4.40]	0.856
	Secondary	2.68 [1.63–4.40]	<0.001	3.07 [1.05–8.95]	0.040	0.75 [0.24–2.31]	0.614
	Higher	27.38 [3.34–224.40]	0.002	6.98 [0.66–73.55]	0.106	-(omitted)	
Current Working status	No	1 (ref)		1 (ref)		1 (ref)	
	Yes	1.06 [0.75–1.50]	0.735	1.32 [0.73–2.38]	0.354	0.94 [0.38–2.31]	0.893
Residence	Urban	1 (ref)		1 (ref)		1 (ref)	
	Rural	0.83 [0.51–1.37]	0.468	0.76 [0.40–1.44]	0.399	0.31 [0.12–0.83]	0.019
Wealth	Poorest	1 (ref)		1 (ref)		1 (ref)	
	Poorer	1.51 [0.89–2.55]	0.124	1.39 [0.61–3.18]	0.428	1.53 [0.43–5.49]	0.512
	Middle	2.17 [1.04–4.55]	0.040	2.38 [0.92–6.13]	0.073	2.39 [0.61–9.31]	0.209
	Richer	1.86 [0.80–4.34]	0.152	2.26 [0.77–6.58]	0.136	0.49 [0.07–3.43]	0.469
	Richest	4.04 [1.20–13.57]	0.024	9.15 [2.27–36.92]	0.002	1.22 [0.09–16.98]	0.882
Self-reported Health status	Good	1 (ref)		1 (ref)		1 (ref)	
	Bad	1.20 [0.78–1.86]	0.411	0.84 [0.42–1.68]	0.623	1.14 [0.35–3.76]	0.826
Distance problem to health facility	Not a big problem (≤30 min)	1 (ref)		1 (ref)		1 (ref)	
	Big problem (>30 min)	0.71 [0.46–1.11]	0.138	1.33 [0.72–2.44]	0.364	1.40 [0.63–3.13]	0.410
Total children ever born	No child	1 (ref)		1 (ref)		1 (ref)	
	1–2 children	10.75 [6.81–16.96]	<0.001	1.99 [0.95–4.18]	0.067	3.28 [0.89–12.13]	0.074
	3+ children	12.15 [6.15–24.01], <0.001		1.28 [0.41–4.03], 0.669		0.25 [0.02–3.52], 0.303	
Health insurance	No	1 (ref)		1 (ref)		1 (ref)	
	Yes	4.37 [2.17–8.77]	<0.001	0.56 [0.21–1.47]	0.235	7.21 [1.01–51.29]	0.048
Frequency to Read newspaper	Not at all	1 (ref)		1 (ref)		1 (ref)	
	<1/week	1.30 [0.57–2.92]	0.532	2.91 [1.21–7.03]	0.017	2.11 [0.56–8.02]	0.271
	≥1/week	3.89 [0.93–16.32]	0.064	0.64 [0.09–4.51]	0.657	1 (empty)	
Frequency to Listen radio	Not at all	1 (ref)		1 (ref)		1 (ref)	
	<1/week	0.84 [0.53–1.34]	0.468	0.65 [0.31–1.38]	0.263	1.05 [0.29–3.82]	0.946
	≥1/week	1.45 [0.94–2.24]	0.091	1.23 [0.68–2.22]	0.485	3.21 [1.16–8.88]	0.024
Frequency to Watch TV	Not at all	1 (ref)		1 (ref)		1 (ref)	
	<1/week	0.79 [0.44–1.41]	0.422	0.60 [0.22–1.61]	0.311	1.46 [0.30–7.02]	0.639
	≥1/week	0.94 [0.58–1.54]	0.806	1.05 [0.49–2.25]	0.896	2.76 [1.10–6.90]	0.030
Frequency to Internet use	Not at all	1 (ref)		1 (ref)		1 (ref)	
	<1/week	1.65 [0.72–3.75]	0.235	0.45 [0.13–1.64]	0.229	3.74 [0.94–14.89]	0.062
	≥1/week	0.99 [0.53–1.87]	0.987	1.68 [0.70–4.02]	0.242	2.89 [0.83–10.03]	0.094
	Almost daily	0.87 [0.50–1.51]	0.627	1.02 [0.47–2.19]	0.961	0.82 [0.21–3.23]	0.774
Region	Western	1 (ref)		1 (ref)		1 (ref)	
	Central	1.13 [0.40–3.25]	0.815	0.63 [0.16–2.44]	0.507	0.66 [0.11–3.80]	0.638
	Greater Accra	0.85 [0.31–2.35]	0.748	0.35 [0.08–1.52]	0.163	1 (empty)	
	Volta	2.59 [0.85–7.91]	0.095	1.14 [0.27–4.71]	0.859	0.70 [0.13–3.81]	0.679
	Eastern	3.62 [1.16–11.25]	0.026	1.09 [0.28–4.25]	0.905	1 (empty)	
	Ashanti	1.12 [0.41–3.07]	0.826	0.18 [0.04–0.80]	0.024	1.06 [0.19–5.81]	0.950
	Western North	1.04 [0.34–3.12]	0.950	0.12 [0.02–0.70]	0.018	0.33 [0.03–4.21]	0.393
	Ahafo	0.71 [0.23–2.14]	0.542	0.97 [0.18–5.11]	0.974	0.24 [0.01–4.21]	0.356
	Bono	1.42 [0.47–4.26]	0.534	0.65 [0.12–3.52]	0.615	1.28 [0.11–14.76]	0.835
	Bono East	2.12 [0.74–6.09]	0.160	0.41 [0.06–2.78]	0.359	1.06 [0.10–10.97]	0.961
	Northern	0.58 [0.19–1.76]	0.333	0.34 [0.06–1.99]	0.226	1.12 [0.13–9.86]	0.920
	North-East	1.16 [0.30–4.54]	0.833	0.40 [0.04–3.98]	0.418	1.00 [0.06–15.38]	0.998
	Upper East	1.01 [0.21–4.88]	0.993	0.61 [0.05–7.37]	0.690	1.36 [0.07–25.99]	0.842
	Upper West	1.71 [0.31–9.46]	0.547	0.40 [0.02–7.65]	0.536	0.65 [0.03–15.83]	0.776
	Savannah	1.50 [0.21–10.86]	0.685	0.24 [0.01–5.01]	0.345	0.33 [0.01–9.39]	0.532
	Oti	0.84 [0.11–6.51]	0.862	0.31 [0.02–4.66]	0.392	0.12 [0.01–2.49]	0.156
Model fitness statistic	HIV testing (full)	HIV testing (null)	Breast cancer screening (full)	Breast cancer screening (null)		Cervical cancer screening (full)	Cervical cancer screening (null)
Log-likelihood	−564.678	−742.831	−346.721	−413.889		−128.749	−176.205
Deviance	1,129.355	1,485.662	693.443	827.777		257.497	352.410
McFadden's R²	0.240	–	0.162	–		0.269	–
Community-level variance	0.608	1.224	1.781	2.491		0.825	1.876
AIC	1,221.356	1,489.662	783.443	831.777		333.497	356.410
BIC	1,463.436	1,500.187	1,020.261	842.302		524.501	366.936
ICC	0.156	0.271	0.351	0.431		0.200	0.363
PVC	0.503	–	0.285	–		0.561	–
MOR	7.057	15.999	28.378	47.100		9.739	21.780

AOR, adjusted odds ratio; MOR, median odds ratio; AIC, akaike information criteria; BIC, bayesian information criteria; ICC, inter cluster correlation; PVC, proportional variance change.

In the HIV testing model, women with secondary education (AOR = 2.68; 95% CI: 1.64–4.39; *p* < 0.001) and higher education (AOR = 27.38; 95% CI: 3.23–232.25; *p* = 0.002) had significantly higher odds of testing compared to those without education. Women with one to two children (AOR = 10.75; 95% CI: 6.02–19.19; *p* < 0.001) or three or more (AOR = 12.15; 95% CI: 5.31–27.80; *p* < 0.001) were more likely to have been tested. Belonging to the richest quintile (AOR = 4.04; 95% CI: 1.20–13.66; *p* = 0.024) and having health insurance (AOR = 4.37; 95% CI: 2.10–9.11; *p* < 0.001) also increased odds of testing.

For breast cancer screening, sexual autonomy (AOR = 2.75; 95% CI: 1.27–5.93; *p* = 0.011), secondary education (AOR = 3.07; 95% CI: 1.05–8.96; *p* = 0.040), and belonging to the richest quintile (AOR = 9.15; 95% CI: 2.25–37.28; *p* = 0.002) significantly increased screening likelihood. However, women in Ashanti (AOR = 0.18; 95% CI: 0.04–0.79; *p* = 0.024) and Western North (AOR = 0.12; 95% CI: 0.02–0.66; *p* = 0.018) regions were less likely to be screened.

In the cervical cancer model, sexual autonomy (AOR = 5.45; 95% CI: 1.43–20.73; *p* = 0.013), health insurance (AOR = 7.21; 95% CI: 1.02–50.81; *p* = 0.048), and frequent radio (AOR = 3.21; 95% CI: 1.17–8.83; *p* = 0.024) and television exposure (AOR = 2.76; 95% CI: 1.10–6.90; *p* = 0.030) increased odds of screening, while rural residence reduced it (AOR = 0.31; 95% CI: 0.12–0.80; *p* = 0.019) (Table 3).

### 4.8 Predictive ability of HIV testing, breast cancer and cervical cancer screening models

**Figure 5:**
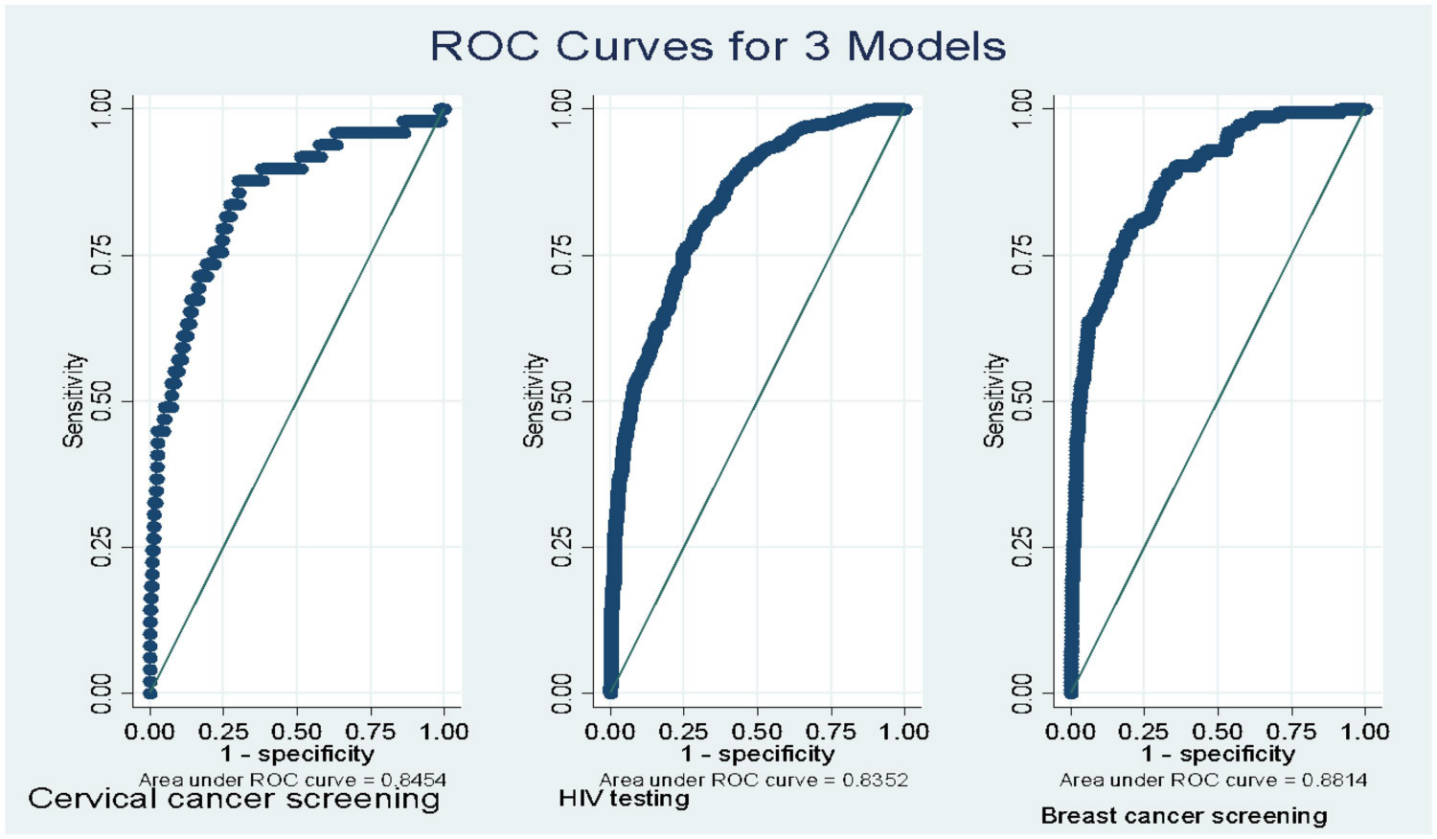
Predictive ability of HIV testing, breast cancer and cervical cancer screening models.

Figure 5 shows the discriminative ability of each screening model assessed using the area under the receiver operating characteristic (ROC) curve (AUC). The HIV testing model demonstrated excellent predictive accuracy with an AUC of 0.835, indicating a strong ability to distinguish between women who had ever tested for HIV and those who had not. Similarly, the cervical cancer screening model achieved an AUC of 0.845, reflecting slightly superior discrimination compared to the HIV testing model. Among the three, the breast cancer screening model showed the highest performance with an AUC of 0.881, suggesting that it most effectively differentiates between women who had and had not undergone breast cancer screening (Figure 5).

### 4.9 Inequality in screening behaviours among young women by wealth status

**Figure 6:**
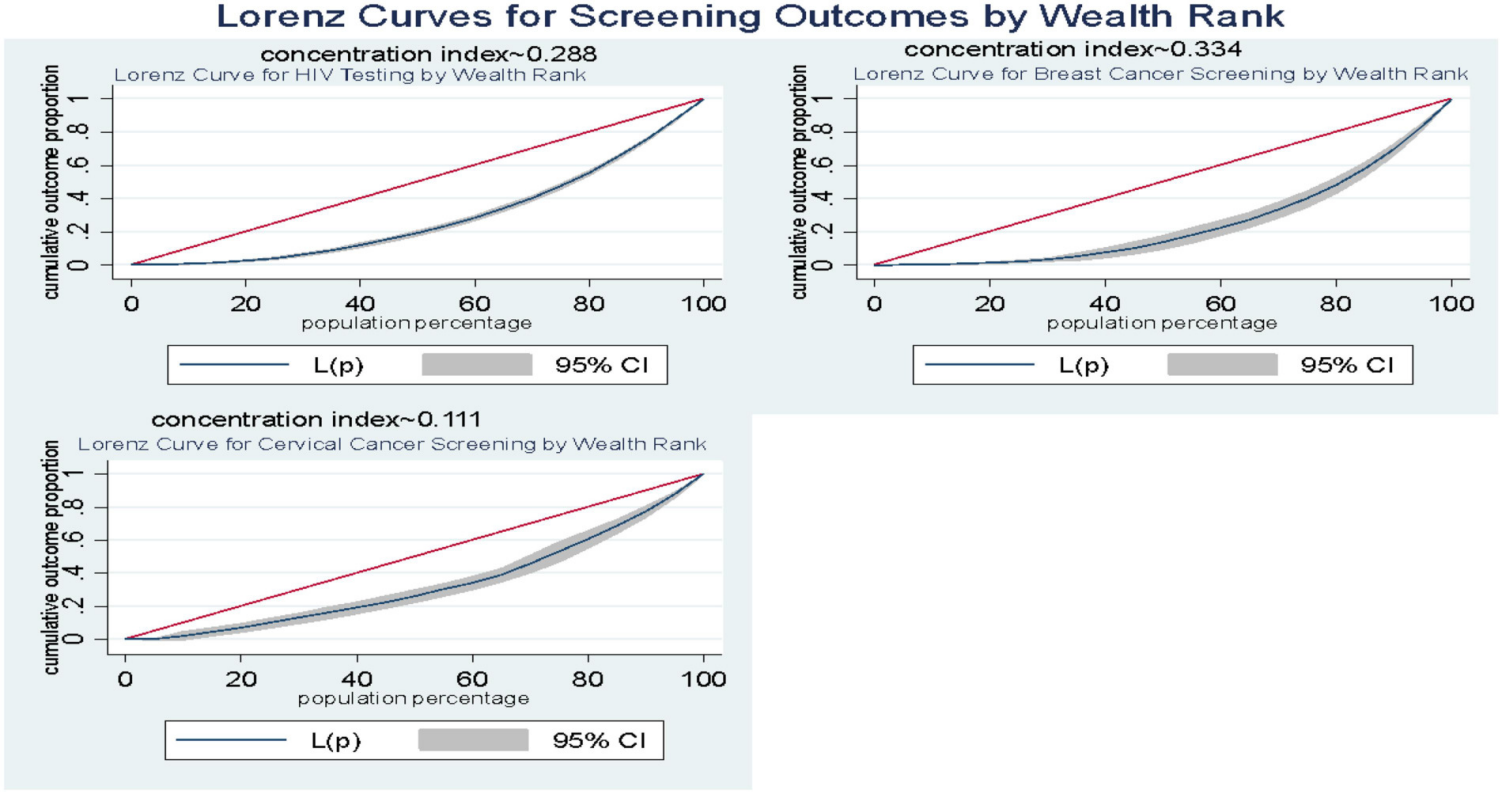
Lorenz curve with concentration index of socioeconomic inequality across various screening outcomes.

Table 4 and Figure 6 present the extent of socioeconomic inequality in HIV testing, breast cancer, and cervical cancer screening among young women in Ghana. The results show marked wealth-related disparities across most screening indicators. HIV testing prevalence increased steadily from 47.1% among the poorest women to 85.0% among the richest, yielding a rich–poor ratio of 1.81 (Table 4) and a positive concentration index (CI = 0.288, *p* < 0.001) (Figure 6). This indicates a pro-rich distribution, meaning HIV testing is disproportionately concentrated among wealthier women. A similar pattern was observed for breast cancer screening, which ranged from 5.8% among the poorest to 32.9% among the richest (Table 4). The rich–poor ratio of 5.72 and CI of 0.334 (*p* < 0.001) suggest even stronger pro-rich inequality in access to breast cancer screening services (Figure 6). In contrast, cervical cancer screening showed relatively low uptake across all wealth groups (ranging from 1.5% among the poorest to 2.3% among the richest) and a nonsignificant concentration index (CI = 0.111, *p* = 0.175) (Figure 6). This indicates that cervical cancer screening remains generally low and equitably distributed, with no clear wealth-related gradient (Table 4).

**Table 4:** Inequality measures across different wealth quintiles for young women's screening behaviours (Wagstaff Conc.Index).

Indicator	Total (%)	Poorest (%)	Poorer (%)	Middle (%)	Richer (%)	Richest (%)	Rich-poor ratio (rpr)	Concentration index (CI)	*p*-value
HIV testing	63.89	47.08	64.19	75.12	68.19	85.02	1.81	0.288	<0.001
Breast cancer screening	12.09	5.75	8.80	13.97	15.53	32.89	5.72	0.334	<0.001
Cervical cancer screening	3.62	1.45	4.20	7.51	2.32	2.30	1.59	0.111	0.175

### 4.10 Subgroup inequality in screening behaviour among young women

Table 5 presents subgroup analyses of socioeconomic inequalities in HIV testing, breast cancer, and cervical cancer screening, stratified by place of residence, education level, and region. The Wagstaff concentration index (CI) quantifies the degree to which each screening behaviour is concentrated among wealthier or poorer women, with positive values indicating pro-rich inequality and negative values showing pro-poor inequality. Across residential subgroups, both HIV testing and breast cancer screening showed pro-rich distributions in urban and rural settings. However, inequalities were significantly higher among rural women for HIV testing (CI = 0.125, *p* = 0.016) and breast cancer screening (CI = 0.173, *p* = 0.0058), suggesting that even within rural populations, screening services remain concentrated among wealthier individuals. Conversely, cervical cancer screening showed a pro-poor gradient in urban areas (CI = −0.184) but a slight pro-rich pattern in rural areas (CI = 0.056), with the within-group difference highly significant (*p* < 0.001).

**Table 5:** Subgroup analysis for socioeconomic inequalities in young women's screening behaviour outcomes (Wagstaff conc. index).

Characteristics	HIV-testing	Breast cancer screening	Cervical cancer screening
Place of Residence			
Urban	0.057	0.251	−0.184
Rural	0.125	0.173	0.056
Sig (*p*-value) difference within place of residence	0.016	0.0058	<0.001
Educational Level			
No education	0.177	0.262	0.329
Primary	0.097	0.147	0.380
Secondary	0.045	0.219	0.005
Higher	0.060	0.660	—
Sig (*p*-value) difference within education	<0.001	<0.001	<0.001
Region			
Western	0.074	0.085	—
Central	0.021	0.171	−0.113
Greater Accra	0.148	0.396	—
Volta	−0.006	0.306	0.772
Eastern	−0.012	−0.110	—
Ashanti	0.007	0.597	−0.200
Western North	−0.012	0.697	0.746
Ahafo	0.056	0.094	0.025
Bono	0.011	−0.307	−0.669
Bono East	0.128	0.690	0.822
Oti	0.091	0.330	0.129
Northern	0.182	0.395	0.509
Savannah	0.201	0.605	—
North-East	0.001	−0.058	0.038
Upper East	0.069	0.167	0.714
Upper West	−0.011	0.370	0.088
Sig (*p*-value) difference within region	<0.001	<0.001	<0.001

Education-related inequalities were also substantial. For HIV testing, the CI declined from 0.177 among women with no education to 0.045 among those with secondary education, implying that inequality reduces with increasing education. In contrast, breast cancer screening inequality was greatest among those with higher education (CI = 0.660, *p* < 0.001), likely reflecting greater awareness and affordability among the educated elite. Cervical cancer screening displayed inconsistent but significant variations across education levels (*p* < 0.001), with the largest pro-rich disparity observed among primary-educated women (CI = 0.380). At the regional level, inequality varied widely. Regions such as Ashanti, Savannah, Bono East, and Western North displayed very high pro-rich inequality (CI ≥ 0.59) for breast cancer screening, while regions like Volta and Upper East showed pro-rich patterns for cervical screening (CIs of 0.772 and 0.714, respectively). Conversely, a few regions such as Bono (breast and cervical screening) and Eastern (HIV and breast screening) demonstrated pro-poor inequality for certain indicators. Overall, the regional variation in inequality was statistically significant across all outcomes (*p* < 0.001) (Table 5).

### 4.11 Equality in screening behaviours across socio-demographic subgroups

Disparities in young women's screening behaviours were unequally distributed across education, region, and place of residence (Table 6). Inequality in cervical cancer screening was most pronounced across geographical regions and urban–rural areas, with Theil index values of 450.0 and 296.0, respectively. Similarly, breast cancer screening showed marked inequity across regions (Theil index = 318.0) and residence (Theil index = 236.0). In contrast, HIV testing exhibited relatively lower but still notable inequality across region (Theil index = 105.0) and education (Theil index = 94.9) (Table 6).

**Table 6:** Equality measures in screening behaviours across education, region, residence (theil index).

Indicator	Region	Residence	Education
HIV testing	105.0	41.6	94.9
Breast cancer screening	318.0	236.0	175.8
Cervical cancer screening	450.0	296.0	51.8

## 5 Discussion

This study provides a comprehensive, nationally representative analysis of the complex interplay between sexual autonomy, socioeconomic factors, and geographic disparities in shaping young Ghanaian women's uptake of essential health screenings. Our findings reveal a landscape of significant inequality, where a woman's ability to control her sexual and reproductive life is linked to her health-seeking behaviour, but this potential is mediated by deep-seated regional and economic disparities.

We found that 77.2% of young, partnered women in Ghana reported moderate to high levels of sexual autonomy. However, this national figure masks profound sub-national variations, with a clear spatial clustering forming a high-autonomy belt in the southern and middle regions (e.g., Eastern, Volta, Bono: 83%–90%) and a low-autonomy cluster in the northern regions (e.g., Northern, North-East, Savannah: 10%–65%). Our national prevalence is higher than the 51.7%—52.8% recently reported in other Ghanaian studies (2, 39) but aligns more closely with pooled sub-Saharan African estimates of 73.0% (5). This discrepancy can be attributed to several factors. First, our study focused specifically on young women (15–24), a demographic that may be increasingly exposed to modern ideas of gender equality and reproductive rights through education and media, unlike studies covering all reproductive age groups. Second, the specific indicators used to construct the autonomy index can vary, influencing the final prevalence. The stark north-south gradient we observed is consistent with the well-documented developmental divide in Ghana, where southern regions benefit from greater infrastructure, educational attainment, and economic opportunities (43). This pattern echoes findings from Ethiopia, where regional disparities in women's decision-making autonomy were also pronounced (11). Furthermore, studies across Africa consistently identify education, media exposure, and wealth as key multi-level predictors of autonomy (2, 21), which aligns with our spatial findings of high autonomy clustering in more developed regions.

Thus, national policies promoting gender equality must be more localized. In high-autonomy southern regions, programs can focus on sustaining and building upon existing empowerment. In the northern belt, interventions must be more fundamental, addressing root causes like girls' education, economic opportunities for women, and community-level dialogues to challenge norms that restrict female sexual agency, like strategies suggested by Kassahun & Zewdie (11). Also, sexual and reproductive health education programs, particularly in low-autonomy regions, should explicitly incorporate modules on negotiation skills, consent, and rights within relationships, moving beyond biological knowledge alone.

A central finding of this study is the differential association between sexual autonomy and screening types. Sexual autonomy was strongly associated with and likely facilitates both breast cancer screening and cervical cancer screening, even after controlling for wealth, education, and residence. In contrast, it was not a significant predictor for HIV testing. The link between autonomy and cancer screening is logical and supported by the literature. Breast and cervical cancer screening are often proactive, voluntary health-seeking behaviours. A woman with greater autonomy is more likely to prioritize her health, have the confidence to seek services for a non-acute condition, and overcome barriers like embarrassment or spousal disapproval (12, 21). Our finding that autonomy increased the odds of cervical screening five-fold is particularly striking and suggests that empowerment may be a crucial lever to overcome the well-documented barriers of fear, shame, and cultural sensitivity surrounding gynaecological examinations (13, 27).

The non-significant association with HIV testing, however, reveals a differing pattern. This is likely because HIV testing for young, partnered women in Ghana is often not a voluntary, autonomy-driven decision but a routine, and sometimes obligatory component of antenatal care (ANC). This is corroborated by our data showing that parity was the strongest predictor of HIV testing. This aligns with studies highlighting the success of prevention of mother-to-child transmission programs in institutionalizing HIV testing within ANC (8, 28, 40). Essuman et al. (8) specifically noted that factors like ANC attendance were key drivers of HIV testing among young Ghanaian women. Our findings extend Andersen's model by demonstrating that enabling factors (wealth, insurance) may be necessary but insufficient without women's agency (autonomy) and supportive community contexts. Further, the differential predictive performance across models (AUC: breast 0.881 vs. cervical 0.845) suggests that breast cancer screening may be more strongly determined by measured factors, while cervical screening involves substantial unmeasured barriers (stigma, cultural factors).

Thus, while autonomy drives voluntary screening, system-integrated testing can achieve high coverage even without it. Hence, cancer prevention campaigns should actively frame screening as an act of self-care and empowerment. These campaigns can partner with women's groups and use empowering messaging to appeal to autonomous women and inspire others. Also, the successful integration of HIV testing into ANC provides a powerful model. Policymakers should urgently accelerate plans to integrate cervical cancer screening into routine ANC and post-natal care services, thereby capturing a captive audience of women who may not otherwise seek screening proactively, as recommended by Mantula et al. (27).

Our inequality analysis uncovered a tiered system of access. HIV testing and breast cancer screening exhibited significant pro-rich inequality (CI = 0.288 and 0.334, respectively), with the richest women having 1.8 and 5.7 times the uptake than the poorest. Cervical cancer screening, meanwhile, was uniformly low across wealth quintiles (CI = 0.111, *p* > 0.05). Spatially, the maps reinforced this, showing screening hotspots in the more developed South (e.g., Eastern, Volta, Central for HIV; Volta, Western for breast cancer) and vast cold spots in the North. The pro-rich inequality in HIV and breast cancer screening is a consistent theme in Ghana and across sub-Saharan Africa, reflecting the cost (direct and indirect) of accessing health services and the correlation between wealth and health literacy (7, 10).

The extreme inequality in breast cancer screening is particularly alarming and suggests it is still perceived as a luxury service. The equitable, albeit catastrophically low, distribution of cervical cancer screening is an equality of deprivation. This finding contrasts with studies in other settings that often find pro-rich disparities (41), but it highlights that in Ghana, barriers like a near-total lack of awareness, extreme scarcity of services, and cultural fears are so universal that they suppress demand across all socioeconomic strata (42). Our spatial findings of a north-south divide are consistent with assessments of maternal health services in northern Ghana, which documented critical gaps in infrastructure and human resources (23). The finding that inequality was sometimes more pronounced within rural areas highlights that poverty and marginalization have layered effects, as even among disadvantaged populations, the least wealthy are further left behind. For HIV and breast cancer screening, targeted subsidies, vouchers, and strengthened national health insurance coverage for preventive services are crucial to level the playing field for poorer women, addressing the financing burdens identified by Macha et al. (10).

Nonetheless, the sub-4% screening rate demands an emergency response. Policy must focus on massive public awareness campaigns to generate demand, combined with rapid scale-up of service availability through primary health centres and mobile clinics, especially in rural and northern regions, as suggested by strategies in Zimbabwe (27). Also, the spatial maps provide an explicit blueprint for the Ghana Health Service, in that; regions identified as cold spots must be prioritized for the deployment of screening equipment, trained personnel, and community-based outreach programs to bridge the geographic disparities highlighted by Langa et al. (42).

Education and media exposure were consistent positive predictors of screening. The role of education and media in increasing health knowledge and shaping positive attitudes is well-established (14, 15). Our finding that radio and TV exposure predicted cervical cancer screening shows their value as channels for health promotion in Ghana, a finding echoed by Enyan et al. (15) in their study of Muslim women. The substantial contextual effect captured by the MOR is a critical insight. It suggests that factors like community norms, the density of health facilities, and the attitudes of local health workers create an environment that either facilitates or hinders screening. This aligns with the concept of clustering of health behaviours and highlights the limitations of interventions that target individuals without changing the community context. This is particularly relevant for cervical cancer, where cultural and religious sensitivities, as noted by Abubakari et al. (13) and Enyan et al. (15), can create a community-wide barrier.

Thus, national health education campaigns should strategically use radio and television, with content tailored to different regional languages and cultural contexts. Entertainment education and talk shows can be effective formats for disseminating messages about screening and normalizing discussions around women's health. Beyond individual awareness, policies should foster enabling environments. This includes training community health workers to promote screening, engaging local leaders and male partners to build supportive social norms, and ensuring that local health facilities are youth-friendly and non-stigmatizing, thereby addressing the multi-level barriers identified in our analysis and supported by Solanke et al. (21).

## 6 Strengths and limitations

This study's major strength lies in its use of nationally representative data, which ensures generalizability of findings to young women across all 16 regions of Ghana. Also, the integration of spatial mapping, multilevel modelling, and inequality decomposition techniques, provided a comprehensive understanding of how sexual autonomy and sociodemographic factors influence women's participation in HIV, breast, and cervical cancer screening. The use of spatial visualization and concentration indices helped identify regional clusters and quantify socioeconomic disparities, while the application of sampling weights and model validation enhanced reliability. Also, our findings challenge purely economic explanations of health-seeking behaviours, contributing additional insights into healthcare disparities, particularly among disadvantaged populations. However, several limitations must be acknowledged. The cross-sectional design limits causal inference, and reliance on self-reported data may introduce recall or social desirability bias. The measure of sexual autonomy, though widely used, may not capture its full cultural and contextual dimensions. Unmeasured community-level factors such as local health promotion activities or service accessibility may also contribute to residual confounding. Despite these limitations, the study provides robust and policy-relevant insights into regional and socioeconomic inequalities in young women's screening behaviours and sexual autonomy in Ghana.

## 7 Conclusions

This study revealed significant geographical and socioeconomic disparities in young women's healthcare screening behaviours and sexual autonomy across Ghana. While HIV testing coverage was relatively high, participation in breast cancer and, particularly, cervical cancer screening remained remarkably low, with marked inequalities favouring wealthier and urban women. Spatial analyses demonstrated distinct north–south clustering, where higher screening uptake and sexual autonomy were concentrated in southern regions, and lower levels were observed in the northern belt. These disparities reveal persistent structural and contextual barriers such as limited access to screening facilities, low health literacy, and socioeconomic disadvantage that hinder equitable participation in preventive health services. Further, sexual autonomy emerged as a strong predictor of women's screening behaviour, highlighting the critical role of empowerment and informed decision-making in promoting preventive health practices. Thus, addressing these inequities requires a comprehensive policy approach that integrates women's health services into community-based platforms, enhances health education, and strengthens gender equity initiatives. Also, expanding access through CHPS compounds, mobile screening units, and digital health promotion campaigns could help bridge existing gaps. Ultimately, ensuring equitable access to screening and strengthening sexual autonomy among young women are essential for achieving Ghana's commitments to Universal Health Coverage and Sustainable Development Goal 3 on good health and well-being.

## Data Availability

Publicly available datasets were analyzed in this study. This data can be found here: https://dhsprogram.com.
