# Supplementary material for: Young women's healthcare screening behaviours and sexual autonomy in Ghana: a spatial distribution and socioeconomic inequality analysis of a large population-based survey
Source: Front Reprod Health. 2026 Feb 9;8:1751165. doi: 10.3389/frph.2026.1751165 (PMC12926498; doi:10.3389/frph.2026.1751165)
Supplement: Supplementary file 1 [file Table1.docx]

Supplementary file 6

| Table 6: Equality measures in screening behaviours across education, region, residence (Theil index) | | | |
| --- | --- | --- | --- |
| Indicator | region | residence | education |
| HIV testing | 105.0 | 41.6 | 94.9 |
| Breast cancer screening | 318.0 | 236.0 | 175.8 |
| Cervical cancer screening | 450.0 | 296.0 | 51.8 |
