# Supplementary material for: Young women's healthcare screening behaviours and sexual autonomy in Ghana: a spatial distribution and socioeconomic inequality analysis of a large population-based survey
Source: Front Reprod Health. 2026 Feb 9;8:1751165. doi: 10.3389/frph.2026.1751165 (PMC12926498; doi:10.3389/frph.2026.1751165)
Supplement: Supplementary file 2 [file Table2.docx]

Supplementary file 5

Table 5: Subgroup analysis for socioeconomic inequalities in young women's screening behaviour outcomes (Wagstaff conc. index)

| Characteristics | HIV-testing | Breast cancer screening | Cervical cancer screening |
| --- | --- | --- | --- |
| Place of Residence |  |  |  |
| Urban | 0.057 | 0.251 | −0.184 |
| Rural | 0.125 | 0.173 | 0.056 |
| Sig (p-value) difference within place of residence | 0.016 * | 0.0058 ** | <0.001 *** |
| Educational Level |  |  |  |
| No education | 0.177 | 0.262 | 0.329 |
| Primary | 0.097 | 0.147 | 0.380 |
| Secondary | 0.045 | 0.219 | 0.005 |
| Higher | 0.060 | 0.660 | — |
| Sig (p-value) difference within education | <0.001*** | <0.001*** | <0.001 *** |
| Region |  |  |  |
| Western | 0.074 | 0.085 | — |
| Central | 0.021 | 0.171 | −0.113 |
| Greater Accra | 0.148 | 0.396 | — |
| Volta | −0.006 | 0.306 | 0.772 |
| Eastern | −0.012 | −0.110 | — |
| Ashanti | 0.007 | 0.597 | −0.200 |
| Western North | −0.012 | 0.697 | 0.746 |
| Ahafo | 0.056 | 0.094 | 0.025 |
| Bono | 0.011 | −0.307 | −0.669 |
| Bono East | 0.128 | 0.690 | 0.822 |
| Oti | 0.091 | 0.330 | 0.129 |
| Northern | 0.182 | 0.395 | 0.509 |
| Savannah | 0.201 | 0.605 | — |
| North-East | 0.001 | −0.058 | 0.038 |
| Upper East | 0.069 | 0.167 | 0.714 |
| Upper West | −0.011 | 0.370 | 0.088 |
| Sig (p-value) difference within region | <0.001*** | <0.001 *** | <0.001 *** |

***p<0.001, **p<0.01, *p<0.05
