# Supplementary material for: Young women's healthcare screening behaviours and sexual autonomy in Ghana: a spatial distribution and socioeconomic inequality analysis of a large population-based survey
Source: Front Reprod Health. 2026 Feb 9;8:1751165. doi: 10.3389/frph.2026.1751165 (PMC12926498; doi:10.3389/frph.2026.1751165)
Supplement: Supplementary file 3 [file Table3.docx]

Supplementary file 4

***p<0.001

| Table 4: Inequality measures across different wealth quintiles for young women's screening behaviours (Wagstaff Conc.Index) | | | | | | | | | |
| --- | --- | --- | --- | --- | --- | --- | --- | --- | --- |
| Indicator | **Total (%)** | **Poorest (%)** | **Poorer (%)** | **Middle (%)** | **Richer (%)** | **Richest (%)** | **Rich-poor ratio (rpr)** | **Concentration index (CI)** | **p-value** |
| HIV testing | 63.89 | 47.08 | 64.19 | 75.12 | 68.19 | 85.02 | 1.81 | 0.288 | <0.001*** |
| Breast cancer screening | 12.09 | 5.75 | 8.80 | 13.97 | 15.53 | 32.89 | 5.72 | 0.334 | <0.001*** |
| Cervical cancer screening | 3.62 | 1.45 | 4.20 | 7.51 | 2.32 | 2.30 | 1.59 | 0.111 | 0.175 |
