# Supplementary material for: Young women's healthcare screening behaviours and sexual autonomy in Ghana: a spatial distribution and socioeconomic inequality analysis of a large population-based survey
Source: Front Reprod Health. 2026 Feb 9;8:1751165. doi: 10.3389/frph.2026.1751165 (PMC12926498; doi:10.3389/frph.2026.1751165)
Supplement: Supplementary file 4 [file Table4.docx]

Supplementary file 3


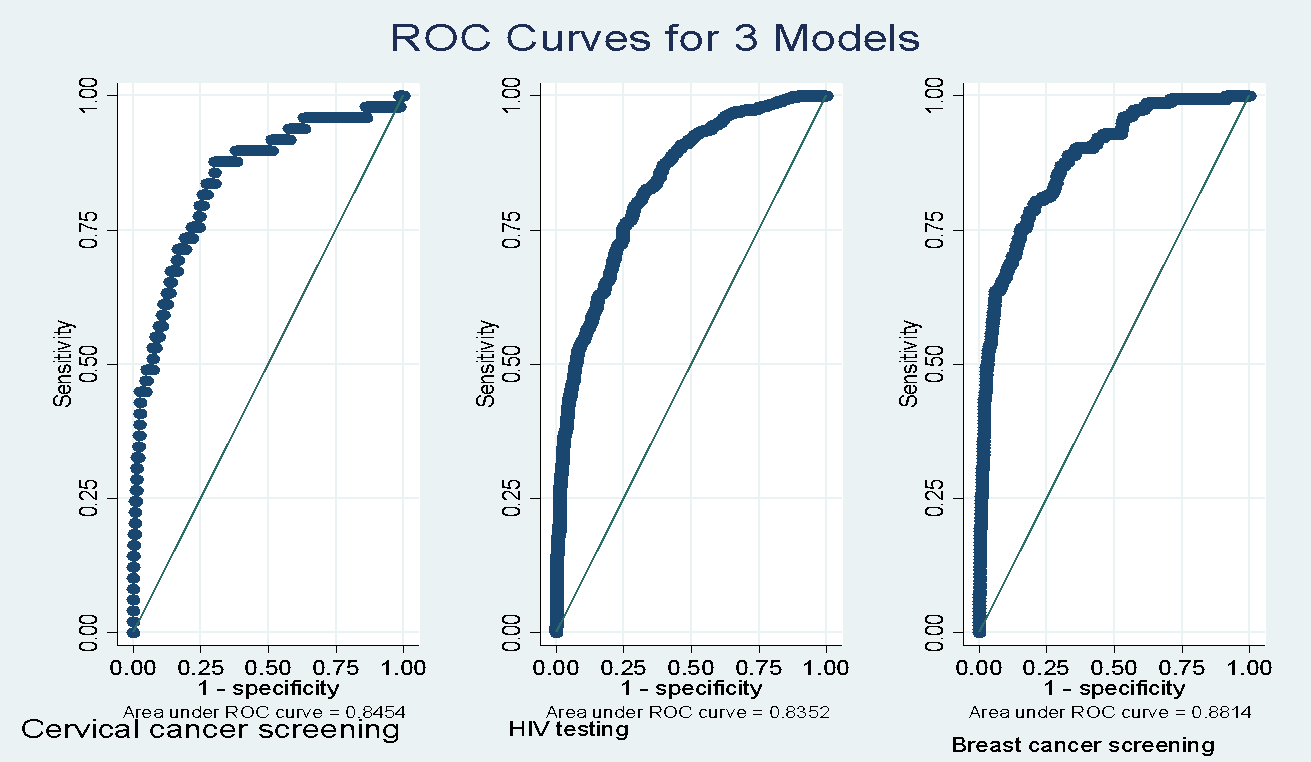


Figure 1: Predictive ability of HIV testing, breast cancer and cervical cancer screening models
