# Supplementary material for: Young women's healthcare screening behaviours and sexual autonomy in Ghana: a spatial distribution and socioeconomic inequality analysis of a large population-based survey
Source: Front Reprod Health. 2026 Feb 9;8:1751165. doi: 10.3389/frph.2026.1751165 (PMC12926498; doi:10.3389/frph.2026.1751165)
Supplement: Supplementary file 5 [file Table5.docx]

Supplementary file 2

Table 3: Multivariable multilevel mixed effects model of predictors of screening practices among young women in Ghana

| Predictor | Category | HIV Testing AOR [95% CI], p | | Breast Cancer Screening AOR [95% CI], p | Cervical Cancer Screening AOR [95% CI], p | |
| --- | --- | --- | --- | --- | --- | --- |
| Sexual autonomy | Low/No | 1 (ref) | | 1 (ref) | 1 (ref) | |
|  | Moderate/High | 1.24 [0.79–1.95], 0.358 | | 2.75 [1.26–5.97], 0.011* | 5.45 [1.44–20.67], 0.013* | |
| Contraception Method | No method | 1 (ref) | | 1 (ref) | 1 (ref) | |
|  | Hormonal | 0.72 [0.48–1.08], 0.114 | | 1.67 [0.95–2.93], 0.074 | 0.39 [0.14–1.11], 0.079 | |
|  | Non-hormonal | 0.63 [0.37–1.08], 0.094 | | 1.11 [0.52–2.39], 0.788 | 0.25 [0.08–0.85], 0.026* | |
| Age | 15–19 | 1 (ref) | | 1 (ref) | 1 (ref) | |
|  | 20–24 | 1.21 [0.79–1.86], 0.383 | | 2.21 [0.93–5.24], 0.073 | 2.29 [0.62–8.49], 0.217 | |
| Education | No education | 1 (ref) | | 1 (ref) | 1 (ref) | |
|  | Primary | 1.41 [0.82–2.44], 0.212 | | 2.72 [0.85–8.66], 0.090 | 1.13 [0.29–4.40], 0.856 | |
|  | Secondary | 2.68 [1.63–4.40], 0.000*** | | 3.07 [1.05–8.95], 0.040* | 0.75 [0.24–2.31], 0.614 | |
|  | Higher | 27.38 [3.34–224.40], 0.002** | | 6.98 [0.66–73.55], 0.106 | - (omitted) | |
| Current Working status | No | 1 (ref) | | 1 (ref) | 1 (ref) | |
|  | Yes | 1.06 [0.75–1.50], 0.735 | | 1.32 [0.73–2.38], 0.354 | 0.94 [0.38–2.31], 0.893 | |
| Residence | Urban | 1 (ref) | | 1 (ref) | 1 (ref) | |
|  | Rural | 0.83 [0.51–1.37], 0.468 | | 0.76 [0.40–1.44], 0.399 | 0.31 [0.12–0.83], 0.019* | |
| Wealth | Poorest | 1 (ref) | | 1 (ref) | 1 (ref) | |
|  | Poorer | 1.51 [0.89–2.55], 0.124 | | 1.39 [0.61–3.18], 0.428 | 1.53 [0.43–5.49], 0.512 | |
|  | Middle | 2.17 [1.04–4.55], 0.040* | | 2.38 [0.92–6.13], 0.073 | 2.39 [0.61–9.31], 0.209 | |
|  | Richer | 1.86 [0.80–4.34], 0.152 | | 2.26 [0.77–6.58], 0.136 | 0.49 [0.07–3.43], 0.469 | |
|  | Richest | 4.04 [1.20–13.57], 0.024* | | 9.15 [2.27–36.92], 0.002** | 1.22 [0.09–16.98], 0.882 | |
| Self-reported Health status | Good | 1 (ref) | | 1 (ref) | 1 (ref) | |
|  | Bad | 1.20 [0.78–1.86], 0.411 | | 0.84 [0.42–1.68], 0.623 | 1.14 [0.35–3.76], 0.826 | |
| Distance problem to health facility | Not a big problem(<=30mins) | 1 (ref) | | 1 (ref) | 1 (ref) | |
|  | Big problem(>30mins) | 0.71 [0.46–1.11], 0.138 | | 1.33 [0.72–2.44], 0.364 | 1.40 [0.63–3.13], 0.410 | |
| Total children ever born | No child | 1 (ref) | | 1 (ref) | 1 (ref) | |
|  | 1–2 children | 10.75 [6.81–16.96], 0.000*** | | 1.99 [0.95–4.18], 0.067 | 3.28 [0.89–12.13], 0.074 | |
|  | 3+ children | 12.15 [6.15–24.01], 0.000*** | | 1.28 [0.41–4.03], 0.669 | 0.25 [0.02–3.52], 0.303 | |
| Health insurance | No | 1 (ref) | | 1 (ref) | 1 (ref) | |
|  | Yes | 4.37 [2.17–8.77], 0.000*** | | 0.56 [0.21–1.47], 0.235 | 7.21 [1.01–51.29], 0.048* | |
| Frequency to Read newspaper | Not at all | 1 (ref) | | 1 (ref) | 1 (ref) | |
|  | <1/week | 1.30 [0.57–2.92], 0.532 | | 2.91 [1.21–7.03], 0.017* | 2.11 [0.56–8.02], 0.271 | |
|  | ≥1/week | 3.89 [0.93–16.32], 0.064 | | 0.64 [0.09–4.51], 0.657 | 1 (empty) | |
| Frequency to Listen radio | Not at all | 1 (ref) | | 1 (ref) | 1 (ref) | |
|  | <1/week | 0.84 [0.53–1.34], 0.468 | | 0.65 [0.31–1.38], 0.263 | 1.05 [0.29–3.82], 0.946 | |
|  | ≥1/week | 1.45 [0.94–2.24], 0.091 | | 1.23 [0.68–2.22], 0.485 | 3.21 [1.16–8.88], 0.024* | |
| Frequency to Watch TV | Not at all | 1 (ref) | | 1 (ref) | 1 (ref) | |
|  | <1/week | 0.79 [0.44–1.41], 0.422 | | 0.60 [0.22–1.61], 0.311 | 1.46 [0.30–7.02], 0.639 | |
|  | ≥1/week | 0.94 [0.58–1.54], 0.806 | | 1.05 [0.49–2.25], 0.896 | 2.76 [1.10–6.90], 0.030* | |
| Frequency to Internet use | Not at all | 1 (ref) | | 1 (ref) | 1 (ref) | |
|  | <1/week | 1.65 [0.72–3.75], 0.235 | | 0.45 [0.13–1.64], 0.229 | 3.74 [0.94–14.89], 0.062 | |
|  | ≥1/week | 0.99 [0.53–1.87], 0.987 | | 1.68 [0.70–4.02], 0.242 | 2.89 [0.83–10.03], 0.094 | |
|  | Almost daily | 0.87 [0.50–1.51], 0.627 | | 1.02 [0.47–2.19], 0.961 | 0.82 [0.21–3.23], 0.774 | |
| Cervical cancer screening | No | 1 (ref) | | - | - | |
|  | Yes | 2.87 [0.96–8.56], 0.058 | | 6.80 [2.38–19.44], 0.000*** | - | |
| Breast cancer screening | No | 1 (ref) | | - | - | |
|  | Yes | 1.91 [0.94–3.86], 0.072 | | - | - | |
| Region | Western | 1 (ref) | | 1 (ref) | 1 (ref) | |
|  | Central | 1.13 [0.40–3.25], 0.815 | | 0.63 [0.16–2.44], 0.507 | 0.66 [0.11–3.80], 0.638 | |
|  | Greater Accra | 0.85 [0.31–2.35], 0.748 | | 0.35 [0.08–1.52], 0.163 | 1 (empty) | |
|  | Volta | 2.59 [0.85–7.91], 0.095 | | 1.14 [0.27–4.71], 0.859 | 0.70 [0.13–3.81], 0.679 | |
|  | Eastern | 3.62 [1.16–11.25], 0.026* | | 1.09 [0.28–4.25], 0.905 | 1 (empty) | |
|  | Ashanti | 1.12 [0.41–3.07], 0.826 | | 0.18 [0.04–0.80], 0.024* | 1.06 [0.19–5.81], 0.950 | |
|  | Western North | 1.04 [0.34–3.12], 0.950 | | 0.12 [0.02–0.70], 0.018* | 0.33 [0.03–4.21], 0.393 | |
|  | Ahafo | 0.71 [0.23–2.14], 0.542 | | 0.97 [0.18–5.11], 0.974 | 0.24 [0.01–4.21], 0.356 | |
|  | Bono | 1.42 [0.47–4.26], 0.534 | | 0.65 [0.12–3.52], 0.615 | 1.28 [0.11–14.76], 0.835 | |
|  | Bono East | 2.12 [0.74–6.09], 0.160 | | 0.41 [0.06–2.78], 0.359 | 1.06 [0.10–10.97], 0.961 | |
|  | Northern | 0.58 [0.19–1.76], 0.333 | | 0.34 [0.06–1.99], 0.226 | 1.12 [0.13–9.86], 0.920 | |
|  | North-East | 1.16 [0.30–4.54], 0.833 | | 0.40 [0.04–3.98], 0.418 | 1.00 [0.06–15.38], 0.998 | |
|  | Upper East | 1.01 [0.21–4.88], 0.993 | | 0.61 [0.05–7.37], 0.690 | 1.36 [0.07–25.99], 0.842 | |
|  | Upper West | 1.71 [0.31–9.46], 0.547 | | 0.40 [0.02–7.65], 0.536 | 0.65 [0.03–15.83], 0.776 | |
|  | Savannah | 1.50 [0.21–10.86], 0.685 | | 0.24 [0.01–5.01], 0.345 | 0.33 [0.01–9.39], 0.532 | |
|  | Oti | 0.84 [0.11–6.51], 0.862 | | 0.31 [0.02–4.66], 0.392 | 0.12 [0.01–2.49], 0.156 | |
| Model Fitness Statistic | **HIV Testing (Full)** | **HIV Testing (Null)** | **Breast Cancer Screening (Full)** | **Breast Cancer Screening (Null)** | **Cervical Cancer Screening (Full)** | **Cervical Cancer Screening (Null)** |
| Log-likelihood | -564.678 | -742.831 | -346.721 | -413.889 | -128.749 | -176.205 |
| Deviance | 1,129.355 | 1,485.662 | 693.443 | 827.777 | 257.497 | 352.410 |
| McFadden's R² | 0.240 | - | 0.162 | - | 0.269 | - |
| Community-level variance | 0.608 | 1.224 | 1.781 | 2.491 | 0.825 | 1.876 |
| AIC | 1,221.356 | 1,489.662 | 783.443 | 831.777 | 333.497 | 356.410 |
| BIC | 1,463.436 | 1,500.187 | 1,020.261 | 842.302 | 524.501 | 366.936 |
| ICC | 0.156 | 0.271 | 0.351 | 0.431 | 0.200 | 0.363 |
| PVC | 0.503 | - | 0.285 | - | 0.561 | - |
| MOR | 7.057 | 15.999 | 28.378 | 47.100 | 9.739 | 21.780 |

***p<0.001, **p<0.01, *p<0.05, AOR=Adjusted Odds Ratio, MOR=Median Odds Ratio, AIC=Akaike Information criteria, BIC=Bayesian Information criteria, ICC=Inter Cluster Correlation, PVC=Proportional Variance Change
