# Supplementary material for: Young women's healthcare screening behaviours and sexual autonomy in Ghana: a spatial distribution and socioeconomic inequality analysis of a large population-based survey
Source: Front Reprod Health. 2026 Feb 9;8:1751165. doi: 10.3389/frph.2026.1751165 (PMC12926498; doi:10.3389/frph.2026.1751165)
Supplement: Supplementary file 6 [file Table6.docx]

Supplementary file 1

Table 2: Weighted Percentage Distribution of Background Characteristics among Women by Screening Outcomes

| Variable | Category | HIV Testing (%) | Breast Cancer Screening (%) | Cervical Cancer Screening (%) |
| --- | --- | --- | --- | --- |
| Sexual Autonomy | Low/No autonomy | 18.6 | 7.2 | 5.2 |
|  | Moderate/High autonomy | 81.4 | 92.8 | 94.8 |
| Method Use | No method | 60.3 | 52.1 | 77.1 |
|  | Hormonal method | 27.4 | 32.3 | 14.4 |
|  | Non-hormonal method | 12.3 | 15.5 | 8.5 |
| Age Group (years) | 15–19 | 12.2 | 7.3 | 8.4 |
|  | 20–24 | 87.8 | 92.7 | 91.6 |
| Education Level | No education | 9.4 | 4.6 | 14.1 |
|  | Primary | 14.1 | 12.5 | 14.2 |
|  | Secondary | 75.1 | 80.5 | 71.7 |
|  | Higher | 1.4 | 2.4 | — |
| Working Status | Not working | 32.2 | 29.2 | 25.6 |
|  | Working | 67.8 | 70.8 | 74.4 |
| Residence | Urban | 44.6 | 60.1 | 64.3 |
|  | Rural | 55.4 | 39.9 | 35.7 |
| Wealth Quintile | Poorest | 21.4 | 13.8 | 11.7 |
|  | Poorer | 21.8 | 15.8 | 25.1 |
|  | Middle | 26.0 | 25.5 | 45.9 |
|  | Richer | 21.5 | 25.9 | 12.9 |
|  | Richest | 9.3 | 18.9 | 4.4 |
| Self-rated Health | Good | 79.3 | 81.3 | 82.1 |
|  | Bad | 20.7 | 18.7 | 17.9 |
| Distance to Facility | Not a big problem | 77.6 | 77.4 | 76.0 |
|  | Big problem | 22.4 | 22.6 | 24.0 |
| Number of Children | No child | 11.1 | 15.2 | 18.0 |
|  | 1–2 children | 79.6 | 78.6 | 81.2 |
|  | 3 or more children | 9.4 | 6.2 | 0.7 |
| Health Insurance | No | 3.9 | 9.2 | 1.4 |
|  | Yes | 96.1 | 90.8 | 98.6 |
| Reads Newspaper | Not at all | 90.4 | 87.2 | 92.3 |
|  | < Once/week | 7.8 | 11.7 | 7.7 |
|  | ≥ Once/week | 1.8 | 1.2 | — |
| Listens to Radio | Not at all | 37.3 | 36.6 | 23.9 |
|  | < Once/week | 21.9 | 18.7 | 16.8 |
|  | ≥ Once/week | 40.8 | 44.7 | 59.3 |
| Watches TV | Not at all | 26.2 | 17.6 | 12.4 |
|  | < Once/week | 13.4 | 10.7 | 13.9 |
|  | ≥ Once/week | 60.4 | 71.6 | 73.7 |
| Internet Use | Not at all | 62.5 | 51.1 | 51.1 |
|  | < Once/week | 4.6 | 2.8 | 8.7 |
|  | ≥ Once/week | 12.0 | 18.1 | 21.9 |
|  | Almost daily | 20.9 | 27.9 | 18.3 |
| Cervical Cancer Screening | No | 95.1 | 89.1 | — |
|  | Yes | 4.9 | 10.9 | — |
| Breast Cancer Screening | No | 84.5 | — | — |
|  | Yes | 15.5 | — | — |
| Region | Western | 6.2 | 11.0 | — |
|  | Central | 11.4 | 13.5 | 14.4 |
|  | Greater Accra | 8.9 | 12.7 | — |
|  | Volta | 4.9 | 7.7 | 5.2 |
|  | Eastern | 9.0 | 11.1 | — |
|  | Ashanti | 16.7 | 9.3 | 27.4 |
|  | Western North | 2.3 | 0.8 | 0.9 |
|  | Ahafo | 2.0 | 3.3 | 2.5 |
|  | Bono | 2.9 | 1.9 | 1.3 |
|  | Bono East | 4.7 | 2.4 | 3.2 |
|  | Oti | 4.2 | 2.8 | 4.6 |
|  | Northern | 9.0 | 8.7 | 27.5 |
|  | Savannah | 1.3 | 1.2 | — |
|  | North-East | 3.9 | 6.0 | 5.8 |
|  | Upper East | 8.6 | 4.9 | 3.6 |
|  | Upper West | 4.1 | 2.7 | 3.5 |
