# Supplementary material for: Young women's healthcare screening behaviours and sexual autonomy in Ghana: a spatial distribution and socioeconomic inequality analysis of a large population-based survey
Source: Front Reprod Health. 2026 Feb 9;8:1751165. doi: 10.3389/frph.2026.1751165 (PMC12926498; doi:10.3389/frph.2026.1751165)
Supplement: Supplementary file 7 [file Table7.docx]

**Highlights**

- Young women in northern Ghana show lower screening and sexual autonomy compared to southern regions.
- Education, wealth, health insurance, media exposure, and sexual autonomy significantly increase screening uptake.
- Strong wealth and regional inequalities persist in rural northern Ghana
